# Nanocellulose Composites as Smart Devices With Chassis, Light-Directed DNA Storage, Engineered Electronic Properties, and Chip Integration

**DOI:** 10.3389/fbioe.2022.869111

**Published:** 2022-08-08

**Authors:** Elena Bencurova, Sergey Shityakov, Dominik Schaack, Martin Kaltdorf, Edita Sarukhanyan, Alexander Hilgarth, Christin Rath, Sergio Montenegro, Günter Roth, Daniel Lopez, Thomas Dandekar

**Affiliations:** ^1^ Functional Genomics and Systems Biology Group, Department of Bioinformatics, Biocenter, University of Würzburg, Würzburg, Germany; ^2^ Laboratory of Chemoinformatics, Infochemistry Scientific Center, ITMO University, Saint Petersburg, Russia; ^3^ Aerospace Information Technology, University of Würzburg, Würzburg, Germany; ^4^ Laboratory for Microarray Copying, Center for Biological Systems Analysis (ZBSA), University of Freiburg, Freiburg, Germany; ^5^ BioCopy GmbH, Emmendingen, Germany; ^6^ Centro Nacional de Biotecnologia CNB, Universidad Autonoma de Madrid, Madrid, Spain; ^7^ Structural and Computational Biology, European Molecular Biology Laboratory, Heidelberg, Germany

**Keywords:** nanocellulose, DNA storage, light-gated proteins, single-electron transistors, protein chip

## Abstract

The rapid development of green and sustainable materials opens up new possibilities in the field of applied research. Such materials include nanocellulose composites that can integrate many components into composites and provide a good chassis for smart devices. In our study, we evaluate four approaches for turning a nanocellulose composite into an information storage or processing device: 1) nanocellulose can be a suitable carrier material and protect information stored in DNA. 2) Nucleotide-processing enzymes (polymerase and exonuclease) can be controlled by light after fusing them with light-gating domains; nucleotide substrate specificity can be changed by mutation or pH change (read-in and read-out of the information). 3) Semiconductors and electronic capabilities can be achieved: we show that nanocellulose is rendered electronic by iodine treatment replacing silicon including microstructures. Nanocellulose semiconductor properties are measured, and the resulting potential including single-electron transistors (SET) and their properties are modeled. Electric current can also be transported by DNA through G-quadruplex DNA molecules; these as well as classical silicon semiconductors can easily be integrated into the nanocellulose composite. 4) To elaborate upon miniaturization and integration for a smart nanocellulose chip device, we demonstrate pH-sensitive dyes in nanocellulose, nanopore creation, and kinase micropatterning on bacterial membranes as well as digital PCR micro-wells. Future application potential includes nano-3D printing and fast molecular processors (e.g., SETs) integrated with DNA storage and conventional electronics. This would also lead to environment-friendly nanocellulose chips for information processing as well as smart nanocellulose composites for biomedical applications and nano-factories.

## GRAPHICAL ABSTRACT

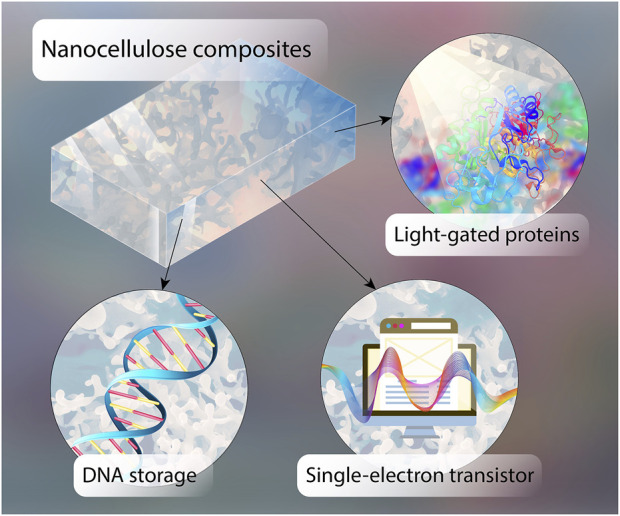

## Introduction

Potential applications using the interaction of nanocellulose with DNA have been investigated for several years. Nanocellulose is a versatile material with several features, such as optical transparency, conductivity, and flexibility. It has various applications, such as packaging material, drug delivery, tissue scaffold, printed electronics, and reinforced polymer composites (Razaq et al., 2011; Thomas et al., 2018; Tao et al., 2020; Jiao et al., 2021) For instance, one approach took advantage of both DNA’s structural compatibility with nanocellulose and its inherent ability for molecular recognition *via* base pairing. By attaching ssDNA oligomers to nanocellulose crystals, it is possible for complementary sequences from oligonucleotides to bind to separate cellulose nanocrystals. They pair with each other creating a nanocellulose/DNA hybrid nanomaterial (Mangalam et al., 2009; Habibi, 2014). In addition to that, nanocellulose-based matrices have been successfully used as an ion-exchange membrane for temporary storage of DNA oligonucleotides (Razaq et al., 2011). However, gradually, nanocellulose composites became ever more attractive and environmentally friendly as multipurpose materials used in medicine, food industry, biotechnology, and engineering (Hoeng et al., 2016; Azeredo et al., 2017; Osorio et al., 2019; de Amorim et al., 2020).

In our study, we explore nanocellulose composite as a smart material. This could be, for instance, a chassis for an information storage device applied preferably to natural, fully degradable components. DNA storage has begun to show large storage potential (Church et al., 2012) for preserving various kinds of data (Goldman et al., 2013) over the course of thousands of years (Grass et al., 2015). Recent major developments had been published recently including the DNA fountain (Erlich and Zielinski, 2017), “DNA-of-things” storage architecture (Koch et al., 2020), and image-based DNA storage systems (Cao et al., 2021). On the other hand, the extraction and decoding time is still challenging. It requires 1 to 3 days, depending on the sequencing technique. Hence, apart from clinical applications such as human genetics/patient samples, it has not yet gained such popularity compared to electronic storage. Here, we evaluate previous concepts (Dandekar et al., 2019) in practice: 1) nanocellulose as a chassis with support, protection, and integration for such a smart device and its components. Nanocellulose was chosen because of its sustainability, it is easy to scale up the production, and it has neither negative nor positive effect on the DNA. 2) We introduce light-gated nucleotide-processing enzymes so that the DNA storage can easily be read and retrieved, accessed, and the information content changed (processed). 3) We show that different electronic properties can be achieved in nanocellulose including semiconductivity and single-electron transistors. 4) The key to unlocking the full potential is to achieve synergies such as light-gated synthesis of DNA wires. This permits adaptive changes in the chip layout. We demonstrate that several useful techniques, including nano-structuring, are readily applied to the nanocellulose composite improving its function. Nevertheless, for high performance, there is still a long way of development to go.

## Materials and Methods

### Preparation of Nanocellulose

As the source of the bacterial nanocellulose, a Kombucha membrane (symbiotic culture of bacteria and yeast) was used (a kind gift of Carmen Aquilar, University of Würzburg). The culture was grown in tea infusion comprising 10 g of black tea (Schwarztee Misching, EDEKA, Germany) and 10% of sucrose (AppliChem, Germany) infused for 10 min into 1 L of boiling water. After cooling to room temperature, 50 ml of media from the previous culture was added to reach the favorable pH for the symbiotic culture. In addition, Kombucha was added. The glass bottle was covered with lightweight paper, and the culture was fermented at room temperature for 30 days. The extraction of nanocellulose was performed using alkaline treatment, acid hydrolysis, and blending.

For the alkaline treatment, the brownish culture was washed 15 times for 10 min in the 1 M NaOH solution using an ultrasonic bath (Sonorex Super, Bandelin, Germany). It was neutralized by washing 10 times for 5 min in distilled water until the membrane reached pH 7.5. After the alkaline treatment, the nanocellulose was ground in a mixer (Princess, 1,000 Wat, 23,000 U/min) for 5 × 2 min and homogenized using glass beads. Dried nanocellulose was obtained by keeping a small amount of the nanocellulose in the desiccator for 48 h.

For the pH-sensitive experiment, 10 μl of malachite green (aqueous solution) was added to 0.5 g of nanocellulose and pH was modified by HCl/NaOH.

### DNA Storage Experiments

For the DNA storage investigations, the text “University of Wuerzburg: Light-gated polymerase” (Supplementary Figure S1) and “Wuerzburg” were encoded into 123 and 26 nt DNA using a DNA writer (Urbano, 2013) and synthesized (Eurofins Genomics, Germany). The encoding schema is given in Table 1. Moreover, 500 picomol of oligonucleotide was subjected to dried nanocellulose and after drying was placed in a sterile closed Petri dish. It was left at room temperature for 2 weeks/2 months/2 years. After these time points, samples were rehydrated in sterile water, and DNA was subcloned to TOPO 2.1 vector (Thermo Fisher Scientific, Germany) and amplified by sequencing primers. Amplicons were sequenced by Sanger sequencing (Eurofins Genomics, Germany). Sequencing was performed in triplicates.

**TABLE 1:** Translation table for DNA storage.

Letter	Code	Letter	Code	Letter	Code	Letter	Code
A	ACT	H	CGT	O	TGT	V	GTA
B	CAT	I	CTG	P	GAG	W	ATG
C	TCA	J	TGC	Q	TAT	X	AGT
D	TAC	K	TCG	R	CAC	Y	GAC
E	CTA	L	ATC	S	TGA	Z	GCA
F	GCT	M	ACA	T	TAG	space	AGC
G	GTC	N	CTC	U	GAT	.	ACG

### Preparation and Analysis of BLUF–GFP Constructs

The *E. coli* strain DH-5α was transformed with a pPK-CMV-F1 vector containing the GFP-encoding gene in the C-terminus (ProKine, Germany) with inserted BLUF domain amplified from the genomic DNA of *E. coli* (forward primer: AAA​AAA​CTG​CAG​AGA​TCT​ATG​CTT​ACC​ACC​CTT​ATT​TAT​CGT​AGC, reverse primer: TTT​TTT​GAG​CTC​TTC​GAA​GCG​AGA​CAG​TAG​TAT​TCA​ATC​GAC​TTT). The transformed *E. coli* was inoculated to 5 ml of the LB medium with ampicillin and cultured overnight at 37 C at 250 rpm. A volume of 1 ml of overnight culture was transferred to two 500 ml flasks with 50 ml of fresh LB medium containing ampicillin and cultures were grown at 37°C to O.D_600_ 0.5. The first bottle was wrapped completely with aluminum foil, and the second was kept under daylight (as a source of blue light). Immediately, 1 mM of isopropyl-β-D-thiogalactopyranoside (IPTG, Sigma-Aldrich, Germany) was added and incubated for 4 h at 30°C. After expression, the cells were analyzed by fluorescent microscopy. Apart from the BLUF–GFP construct, we also constructed other light-activated nucleotide-processing enzymes for the functional assays: LOV–Taq polymerase, BLUF–adenyltransferase, BLUF–T4 kinase, BLUF–Cid I polymerase (including mutated and wild type version and two different BLUF domain constructs), and LOV 2–adenylate kinase (see Supplementary Table S1 for available constructs). Sequences are listed in the Supplementary Material.

### Exonuclease Production and Activity Assay

The BLUF–exonuclease construct was ordered as a synthetic gene (Eurofins Genomics, Germany) and cloned into an expression vector (pQE-30-UA-mCherry-GFP, in-house modified plasmid from Qiagen, United States). The protein was expressed as described above. Purification was performed using the Ni-NTA resin (Merck, Germany), following the manufacturer’s instructions. For the activity assay, 1 µM of Cy-5 hexamer (AAAAAA) was mixed with 10 U of exonuclease I (NEB, Germany) as the positive control, or 1 µl of BLUF–exonuclease I. The mixture was incubated for 30 min at 37°C and then heat-inactivated. Samples were mixed with TBE-Urea sample buffer (Thermo Fisher Scientific, Germany) and resolved on 10% TBE-Urea gel. The gel was scanned by Odyssey (LI-COR, Germany).

### CidI Polymerase Docking

The CidI molecule (PDB: 4FH5 and 4FHX) and ligands (adenine and uracil) were energy-minimized before docking with the help of the Molecular Operating Environment (MOE) software [Molecular Operating Environment (MOE), 2016]. This was carried out with the MMFF94 (Merck Molecular) force field. Protein structure refinement, as well as ligand library preparation, was carried out with the tools of the same software. Molecular docking simulations were performed using GOLD (Jones et al., 1997), MOE (Molecular Operating Environment (MOE), 2016), and AutoDock (Morris et al., 2009).

The AutoDock 4.2.6 (Morris, et al., 2009) software was obtained from the site of “The Scripps Research Institute” (http://autodock.scripps.edu/downloads/autodock-registration/autodock-4-2-download-page/) to perform molecular docking simulations.

The structure of polymerase was protonated, and the rotatable bonds for the ligands were clearly defined. The dimension of the grid box was set as 50 Å × 50 Å × 50 Å for all the docking simulations along with spacing of 0.375 Å. The center of the grid box was placed so that it involved the residues of the active site of the protein and coincided with the center of ligand in the active site.

A Lamarckian genetic algorithm (GA) was applied for all the docking simulations. The orientation, torsions, and position of the drug molecule were set randomly. A total of 50 runs GA were performed for each docking. The final analysis of ligand conformations as well as their interaction profile with a target protein was performed using Chimera software version 1.14 (Pettersen, et al., 2004).

The scoring function is represented by ΔG_bind_—the binding energy difference between the bound and unbound states both for macromolecule and ligand.

$$\Delta G_{bind}=\Delta G_{vdW}+\Delta G_{elec}+\Delta G_{hbond}+\Delta G_{desolv}+\Delta G_{tors},$$

where Δ*G*_*vdW*_ gives the difference in free energy due to van der Waals interactions in the bound and unbound states. Δ*G*_*elec*_ is the energy difference due to electrostatic interactions in the bound and unbound states, Δ*G*_*hbond*_ describes the difference in free energies due to hydrogen bond formation between the unbound and bound states, Δ*G*_*desolv*_ represents the desolvation free energy change in the unbound and bound states, and Δ*G*_*tors*_ is the energy difference of torsional entropy. The standard error for Δ*G* is around 2 kcal/mol (Forli, et al., 2016).

### Sequenase Activity

The pH sensitivity of enzymes was demonstrated on the Sequenase ver. 2.0 (Affymetrix, United States). As the template, the DNA from bacteriophage M13mp18 was used. The reactions were performed as per manufacturer’s instructions. The pH of the reaction was modified by HCl and NaOH.

### T4 Kinase Phosphorylation Activity Assay

This assay followed the methods of Song and Zhao (2009) facilitating optical monitoring of the phosphorylation increase and conformational change of the substrate oligonucleotide. Using the light-gated T4 kinase construct, we observed light-controlled phosphate transfer. The assay for the T4 kinase constructs used the processed fluorescent oligonucleotides (Song and Zhao, 2009), for monitoring their activity; the calculations for the constructs considered cooperative changes following Halabi et al. (2009) and Lee et al. (2008).

### Modeling of the Properties of a Nanocellulose-Based Single-Electron Transistor

The nanocellulose monomer was retrieved from the PubChem database as a 2D structure and converted to the 3D model by the ChemAxon software (Costache et al., 2018; Kim et al., 2021). The nanocellulose (NCL)-based SETs with and without iodine modification (I, I_2_, and I_3_ atoms), consisted of gold (111) nano-electrodes and a subunit of nanocellulose molecule as a central element. These were carried out with the combination NCL-SET, NCL(I)-SET, NCL(I_2_)-SET, and NCL(I_3_)-SET as molecular junctions were constructed by the Atomistix ToolKit suite and simulated using the DFT model available in the ATK software (Smidstrup et al., 2017). To analyze the HOMO (highest occupied molecular orbital) and LUMO (lowest unoccupied molecular orbital) levels of NCL in the presence of the surrounding electrodes, the Molecular Projected Self-Consistent Hamiltonian (MPSH) was performed to obtain the MPSH states by diagonalizing the molecular part of the full self-consistent Hamiltonian (Stokbro et al., 2003).

### Iodine Doping of Nanocellulose

A never-dried bacterial nanocellulose hydrogel was immersed in aqueous iodine solutions of different concentrations at room temperature for 24 h with constant shaking (150 rpm). Iodinated nanocellulose was rinsed with distilled water five times for 5 min to remove the remaining iodine. Measurements of conductivity were performed in triplicate by Laqua Twin (Horiba, Japan).

### Engineered Patterns in a Real Biofilm

Key sensor histidine kinase genes from *B. subtilis* bacteria were artificially deleted (*kinC* and *kinD*). Another experiment utilized spontaneous mutations in the strong biofilm repressor *sinR*, which reinitiate tight interactions and achieve patterning of colonies with biofilm-forming and non-forming regions (right colony). For large-scale active DNA storage, the light-gated and monitoring constructs have to be introduced.

### Membrane Dyes to Monitor Membrane Damage

Nile red-stained bacterial membrane and accumulated in areas where damage has been made.

## Results

### Nanocellulose as Support, Chassis, and Protection for DNA Storage

**FIGURE 1:**
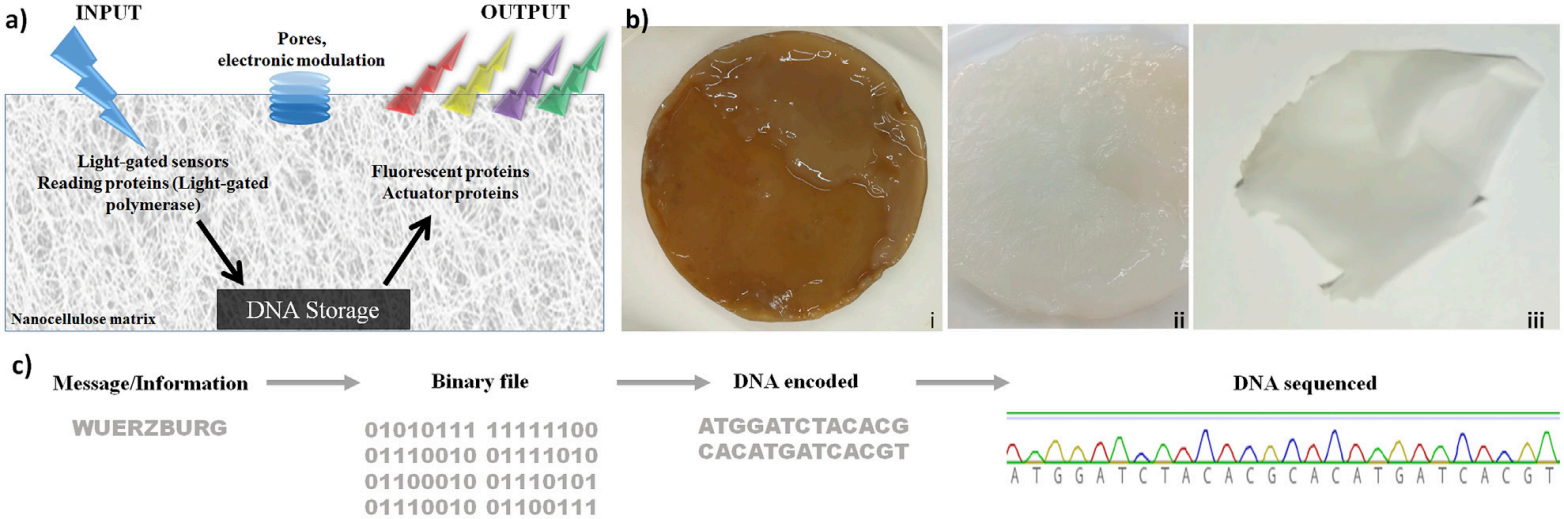
Nanocellulose (NC) as support, chassis, and protection for DNA storage. **(A)** DNA chip concept: after light stimulation, proteins are activated and allowed to read the information stored on the nanocellulose. Subsequently, the actuator proteins read and analyze the processed DNA. **(B)** Production of bacterial nanocellulose **(i)** not purified bacterial nanocellulose from SCOBY (symbiotic culture of bacteria and yeast), **(ii)**: NC purified, and **(iii)** dried. **(C)** DNA storage on NC: the word “Wuerzburg” was encoded by an easy encoding system, where three nucleotides were used to encode one letter. The resulting DNA strand was synthesized and stored on a dried NC film at room temperature. After 2 weeks, 2 months, and 2 years, the DNA was extracted from the NC and sequenced.

These results are shown in Figure 1. The concept is shown in Figure 1A: nanocellulose as chassis for DNA storage, including efficient DNA protection, while processing enzymes as well as electronic modulation are later steps. Production and purification of bacterial nanocellulose (NC) are depicted in Figure 1B. To test storage capabilities, we used different DNA oligonucleotides. Figure 1C shows how the word “Wuerzburg” is encoded with a simple coding schema using three nucleotides per letter. The resulting DNA was synthesized and stored on dried NC at room temperature (Figure 1Biii). After 2 weeks, 2 months, and 2 years, the DNA was retrieved and sequenced by Sanger sequencing (Figure 1C). Sequenced DNA encoded the longer segment (“University of Wuerzburg light-gated polymerase”) stored for 2 years is depicted in Supplementary Figure S1. We noted some change/mutation in the nucleotide sequence but every nucleotide was well preserved. Looking at the long-term storage properties of nanocellulose, we estimated that under ambient conditions the DNA will be stable for at least 10 years. Under cold preservation conditions (freezer at −20 or even −80 ), this time would be much longer. Though this is only based on extrapolation, it suggests for nanocellulose storage in DNA good preservation for many years without any nucleotide errors.

In addition to these concrete results regarding protection, there are also general advantages: 1) the additional protection of DNA against UV radiation (Zhang et al., 2019; Klochko et al., 2021) is a clear advantage of nanocellulose. Whether this leads to as good preservation as in bones (Allentoft et al., 2012) or by vitrification (Grass et al., 2015) remains to be tested. Moreover, several other advantages of nanocellulose have become evident, in particular, 2) nanocellulose provides a versatile composite. Moreover, 3) it can be made transparent, allowing transparent coverage or can protect a display. 4) It has been shown to integrate well and efficiently functional electronic parts, and 5) it is in general an ideal composite host entity, integrating many different materials. It is also worth noting that nanocellulose is more ecologically friendly and easier to produce under standard laboratory conditions than a filter paper (made from normal cellulose). For performant DNA storage besides direct protection of DNA and the excellent properties of nanocellulose as a host material are important. Nanocellulose as a versatile composite is critical so that, for instance, conventional electronics can be directly integrated. Furthermore, a read-out of the storage can be displayed using transparent nanocellulose.

### Integration of DNA Storage for Easy Read-In and Read-Out

**FIGURE 2:**
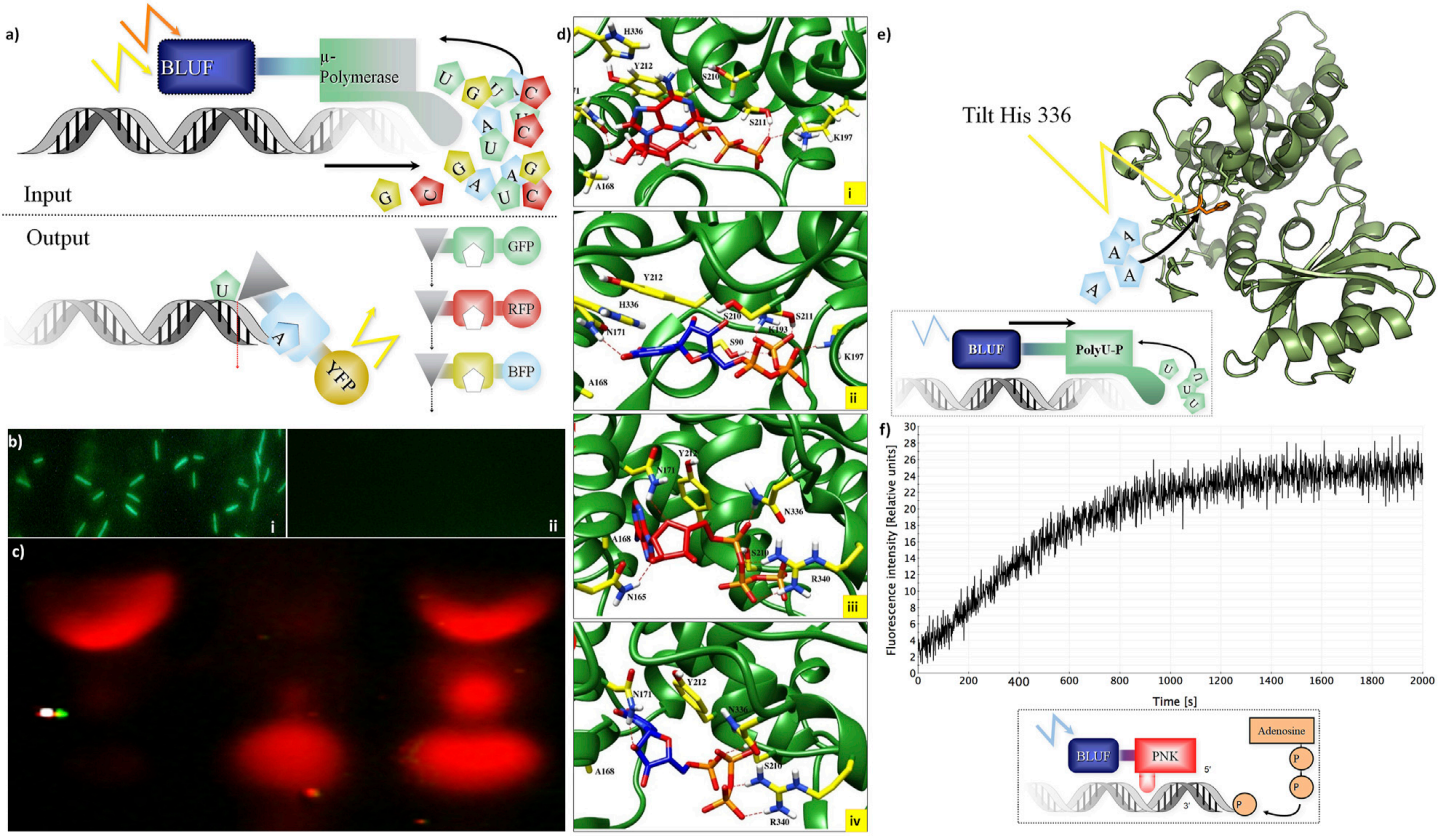
Integration of DNA storage for easy read-in and read-out. **(A)** Concept of light-gated protein. Input (top): μ-DNA polymerase is used to achieve light-gated (BLUF domain fused to μ-DNA polymerase constructs for each nucleotide) and template-free DNA synthesis. Output constructs **(**bottom): light-gated exonuclease constructs (triangles) are fused to specific nucleotide-binding domains (squares) and trigger different fluorescent proteins for read-out. **(B)** Light-gated protein expression *in vivo.* BLUF–GFP construct transfected by bacteria with different expressions of GFP with (Panel **(i)**) and without (Panel **(ii)**) presence of blue light. **(C)** Light-gated exonuclease activity: first line: positive control (only cyanine-5–labeled hexamer loaded on gel), second line: BLUF–exonuclease activity in the dark, third line: BLUF–exonuclease activity with the presence of blue light. **(D)** Modeling: ATP (red) and UTP (blue) at binding site of the Cid1 polymerase. Panel **(i)**: docking of ATP in the wt Cid1 binding pocket. Panel **(ii)** depicted the UTP-Cid1 for the docking to the binding pocket of the wt Cid1 (PDB: 4FH5). In panel **(iii)** (ATP-Cid1 complex) and **(iv)** (UTP-Cid1 complex), the complexes are shown for the case of the H336N mutation (PDB: 4FHX). The histidine 336 does not form any hydrogen bonds with the ligands, whereas N336 is involved in H-bond formation with ATP. Residues S211 and K197 from the binding cavity of the wt. of Cid 1 polymerase (H336) are participating in hydrogen bond formation with ATP, CTP, GTP and UTP. K193 participates in hydrogen bond formation with all triphosphates, except ATP. In the latter N336 and R340 play roles in H-bond formations with ATP, GTP and UTP. N171 forms H-bonds with ATP, CTP, and UTP, but it does not form H-bond with GTP. Moreover, the number of residual contacts for the ATP as well as for the UTP docked to the binding cavity of H336N polymerase is higher compared to the ATP and UTP docked to the native structure. We may assume that mutation of H to asparagine at position 336 could influence the binding mode of ATP as well as UTP. Key amino acid residue composition inside the binding site involved in hydrogen bond formations are highlighted in yellow. Red dashed lines indicate the H-bonds. **(E)** Histidine tilting of PolyU polymerase (structure modeling). Top: a histidine in the PolyU polymerase domain (PDB file shown: 4FH3) determines if ATP or UTP will be accepted for the elongation of the RNA strand. The histidine 336 could be tilted by light to achieve rapid changes in substrate specificity according to user-specified sequences of ATP and UTP. **(F)** T4 Polynucleotide kinase as an example of DNA-processing enzyme. Light-controlled phosphate transfer measurement: assay for T4 kinase DNA elongation constructs using processed fluorescent oligonucleotides for monitoring their activity; construct calculations to predict joined cooperative changes after (Lee et al., 2008; Halabi et al., 2009). Bottom: a sketch of the light-gated protein construct. A BLUF domain (BLUF, blue) is directed by light (blue flash) and stops or activates the T4 polynucleotide kinase (PNK, red) which then transfers a phosphate group from ATP to the end of a polynucleotide **(**example: DNA, shown in grey shape).

For DNA storage allowing active read-in and read-out, we investigated nucleotide-processing enzymes (Figure 2). The concept (Figure 2A) relies on light-gated proteins, allowing them to control their activity by the light of specific wavelengths (e.g., blue light using LOV and BLUF domain as a sensor). “Read-in”: the four DNA nucleotides are incorporated only if the DNA polymerase investigated here is fused to a blue light–harvesting domain (BLUF I/II domain). This activates a polymerase (Figure 2A). The LOV–Taq polymerase (Supplementary Table S1) works quite efficiently, but it can also be a template-free polymerase such as μ-DNA polymerase (Uchiyama et al., 2009). This activation by a cooperative structure change happens only if blue light hits the BLUF protein domain linked to the polymerase.

This concept was tested in practice first in a simpler construct controlling GFP activity by fusing it to a BLUF domain *in vivo* (Figure 2B). This then allows switching on green protein fluorescence only if the construct is exposed to blue light before (top), otherwise not (bottom). However, more interesting is the control of nucleotide-processing activity. “Read-out”: we show light-gated exonuclease activity in Figure 2C. The exonuclease is operating on a Cy-5–labeled DNA hexamer. This demonstrates the concept of read-out from the DNA storage controlled by light. Similarly, we constructed several light-gated constructs (Supplementary Table S1) and different oligonucleotides for each application (Supplementary Table S2). All experiments on read-in and read-out for the different constructs were done each at least in triplicates. We can state that these light-gated constructs (exonuclease, polymerases, and nucleotide processing) work well in an efficient manner. We have thus clear “read-in” and “read-out” of information into the DNA storage.

We next wanted to study whether this is also possible for RNA, and here the Cid1 polymerase has experimentally proven polyU addition activity (Lunde et al., 2012). We tested, in addition, whether the light-gated constructs work on Cid1 polymerase and observed clear dependence of activity if the light was given; there was not any RNA synthesis if no light was present.

Next, we wanted to understand how substrate change can be effected using Cid1 polymerase. For this, specific mutations that change substrate preference from uracil to adenine are known (Lunde et al., 2012). We hence created a light-gated version of the H336N mutated version of the Cid1 polymerase. Substrate specificity can be changed by mutating H336 to A or N. The H336N mutation of Cid1 polymerase was shown by data from experiments to have a preference for ATP instead of UTP (Lunde et al., 2012). Hence, using wild type as well as the mutated version of Cid1 polymerase (made as described in *Materials and Methods*), we can either preferentially add UTP or ATP to the RNA template. In such a PCR-like process of changing the enzyme activated or used, one can, in principle, “write” with RNA. It first adds adenine, then uracil, next adenine, and so on.

The structure details of this change in substrate specificity were further investigated *in silico*.

We investigated different structures and models, thus besides Cid1 polymerase (with several available structures) there is also a PDB file (2Q66) of a yeast poly(A) polymerase with ATP and oligo(A). In result, we compare and show here one PDB structure for the native (4FH5) and one for the mutated Cid1 (4FHX) so that we can complement the experimental data we have on Cid1 polymerase by exactly the corresponding PDB structures. For our analysis, we do not show the RNA substrate in full, but just the nucleotides. Our study was designed to exactly match the already published work of Lunde et al. (2012), in which the authors showed by experiments with nucleotide triphosphates (ATP, UTP, etc) that the H336 in the Cid1 polymerase is the crucial and essential binding site for the ATP binding and recognition. Using the structure of Cid1 polymerase (PDB file 4FH5 for wild type and PDB 4FHX for mutated version) for *in silico* modeling, the cleft of the mutated nucleotide-processing enzyme is compared to the wild type in Figure 2D. Cid1 is compared in a state in which the template and product RNA strands are bound and the active site is open. The conformations of ATPs (Figures 2Di,iii) inside the pocket differ from those of UTPs (Figures 2Dii,iv) when bound to the native structure of Cid1 polymerase versus the mutated H336N (detailed binding interactions in legend). In Table 2, the binding energies for nucleotide triphosphates are compared, in wild-type Cid1 UTP binds best with −9.35 kcal/mol, and mutated H336N prefers ATP (−8.63 kJ/mol) over UTP. H336 is hence critical for substrate specificity. These calculated binding energies give support to what has been seen by the experimental data and are in accordance with the well-known specificity of such polymerases.

**TABLE 2:** Cid1 polymerase substrate binding comparing wild type and H336N mutant.^a^

	ATP	CTP	GTP	UTP
H336	−6.81	−9.12	−8.83	−9.35
H336N	−8.63	−7.78	−7.65	−7.87

a The free energies of binding ΔG (kcal/mol) for the ATP, CTP, GTP, and UTP, from the dockings to the binding cavity of the native Cid1 polymerase as well as to the one with H336N mutation. Detailed methods and standard error estimates are given in *Materials and Methods*.

The histidine 336 is pivotal for specificity (see also experimental data in Yates et al., 2012), and it was recognized as essential ATP binding site (Lunde et al., 2012). Could the histidine be changed in its conformation by light in a similar way as the light-gated domains controlling the enzyme activity? By this, we would change substrate specificity “on the fly” just by a light pulse and without a mutation and hence fast for many nucleotide synthesis steps instead of the slow PCR-type way we indicated above. In principle, this seems to be possible, but as these are challenging experiments to actually achieve this, this was again only investigated *in silico*.

Thus, irradiating the histidine at the binding pocket with its absorption maximum wavelength at 220 nm (as determined by M. O. Iwunze, 2007), such a light pulse, if energetic enough should interfere with the binding pocket. For comparison, Wei et al. (2007) identified a single tryptophan (Trp) residue responsible for loss of binding and biological activity testing this by UV light irradiation in a humanized monoclonal antibody (MAb) against respiratory syncytial virus (RSV). Changing the histidine 336 conformation by UV light (using its 220 nm optimum for histidine light absorption), it would thus allow in principle rapid change of the substrate cleft around the histidine (Figure 2E; structure modeling cartoon). However, whether this can allow rapid change of incorporation of the nucleotides during active polymerization, for example, changing adenine for uracil remains to be determined in future experiments.

A digital addition of individual nucleotides can also be achieved by using nucleotidyltransferases. Here, the light-gated constructs for different wavelengths allow us again to control the activity of the nucleotidyltransferases (e.g., BLUF and LOV, see *Materials and Methods*, or using instead halorhodopsin). Each nucleotide is added and then the transferase is halted. However, this was not yet studied in experiments here.

Finally, we monitored directly activity switches of DNA-processing enzymes after light-gating activation using a BLUF domain. This is shown for T4 polynucleotide kinase, allowing nucleotide processing by phosphorylation modification if activated by blue light (Figure 2F). Moreover, ATP and energized nucleotides are easily integrated into the composite. They can be regenerated using either current or, for example, pH gradient–powered ATP synthetases and/or adding adenylate kinase to buffer the concentrations of all four nucleotides against each other.

**FIGURE 3:**
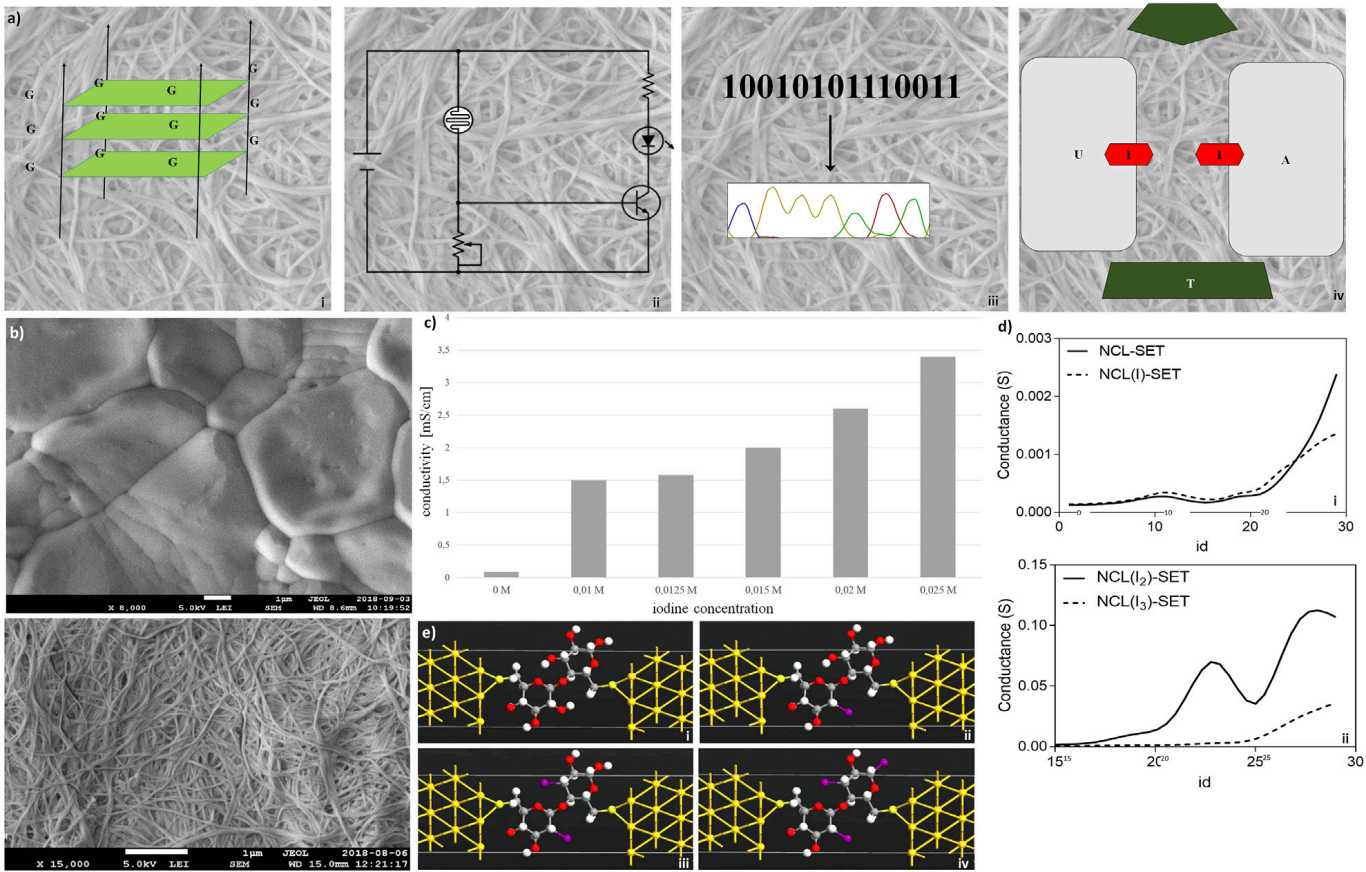
Electronic features of nanocellulose. **(A)** Concept: nanocellulose can replace silicon, for example, introducing electronics and modifying enzymes: **(i)** G-wire quartets, allowing conduction of electric current; **(ii)** condenser/resistor, different printed electronics can be applied; **(iii)** sequencing result, different enzymes and DNA can be attached on the NC; and **(iv)** represents the introducing of SET. **(B)** Nanocellulose becomes conductive with iodine doping (0.025 M iodine) (Panel **(i)**), untreated control is depicted in Panel **(ii)**. **(C)** Measured conductivities for the iodine treated nanocellulose. **(D)** Conductance curves **(i,ii)** calculated for the nanocellulose (NCL)-based SETs with and without iodine modification (I, I_2_, and I_3_ atoms), consisting of gold (111) nano-electrodes and a subunit of nanocellulose molecule as a central element: NCL-SET, NCL(I)-SET, NCL(I_2_)-SET, and NCL(I_3_)-SET as molecular junctions. **(E)** *In silico* molecular illustration of nanocellulose (NCL)-based SETs with and without iodine modification (I, I_2_, and I_3_ atoms): consisting of gold (111) nano-electrodes and a subunit of nanocellulose molecule as a central element. The nanocellulose-based SETs: NCL-SET **(i)**, NCL(I)-SET **(ii)**, NCL(I_2_)-SET **(iii)**, and NCL(I_3_)-SET **(iv)** as molecular junctions were designed using the Virtual NanoLab software.

Electronic features of nanocellulose are investigated in Figure 3. The concept is shown in Figure 3A: nanocellulose replaces silicon and becomes electronic. First of all, nanocellulose can well incorporate or attach nucleic acids such as DNA or RNA after chemical treatment or UV crosslinking. One possibility is to integrate electronic features and electric current. This can be accomplished (Figure 3Ai) through the incorporation of DNA wires such as G-wire quartets (Livshits et al., 2014). However, by direct treatment, nanocellulose may become electronic conductive and act as a capacitor or resistor (Figure 3Aii). Complex treatment is not necessary. We typically used a simple protocol where the aqueous suspension of DNA was directly subjected to the dried nanocellulose and the suspension was subsequently left for drying. As we used double-stranded DNA, no further procedure was necessary such as UV-immobilization or acid treatment. However, activation by acid or other pH change is another method we tested to improve DNA attachment and achieve covalent linkage.

Hence, a sequencing result may either be stored by DNA or even electronically in our nanocellulose composite. For optimal information processing, both types of storage may be used and combined (Figure 3Aiii). For actual electronic parts made from nanocellulose, we suggest and investigated a SET (single-electron transistor) as an attractive molecular electronics device (Figure 3Aiv). The experimental results regarding nanocellulose are shown in the following: iodine treatment makes the nanocellulose conductive. The treated nanocellulose surface is shown by electron microscopy (Figure 3Bi), and the untreated nanocellulose is shown for comparison in Figure 3Bii. The measured conductance in nanocellulose for different concentrations is given in Figure 3C. Nanostructured bridges and connections are readily visible in the electron micrographs of the nanocellulose (iodine treated and untreated samples) and are a natural part of the fine structure of the nanocellulose. However, actual electronic parts would require ultrafine structuring of the nanocellulose surface. This could be efficiently achieved with coverage by photo-paint and UV etching as in conventional semiconductor manufacturing. To estimate the miniaturization potential in this direction for electronic nanocellulose we calculated *in silico* conductance curves for the nanocellulose (NCL)-based SETs with and without iodine modification (I, I_2_, and I_3_ atoms; Figure 3D panel i for 0 and 1 iodine atoms and panel ii for 2 and 3 iodine atoms). Figure 3E shows in detail the dimensions of a SET, exploring maximal miniaturization for nanocellulose-based semiconductors: each SET consists of gold (111) nano-electrodes and a subunit of nanocellulose molecule as a central element: NCL-SET, NCL(I)-SET, NCL(I_2_)-SET, and NCL(I_3_)-SET as molecular junction. The predicted good performance properties of such a nanodevice are furthermore supported by orbital calculations on a nanocellulose SET (Table 3) and supported by the actual conductance data for the iodine-treated nanocellulose.

**TABLE 3:** Nanocellulose single-electron transistor properties.^a^

Nanodevice	HOMO^a^	LUMO^a^	Gap
NCL-SET	−1.18	4.97	6.15
NCL(I)-SET	−1.32	2.77	4.09
NCL(I_2_)-SET	−1.31	2.77	4.08
NCL(I_3_)-SET	−1.18	1.31	2.49

a HOMO (highest occupied molecular orbital) and LUMO (lowest unoccupied molecular orbital) calculations for the NCL-based SETs, with and without iodine modification (I, I_2_, and I_3_ atoms), consisting of gold (111) nano-electrodes and a subunit of nanocellulose molecule as central element (all units are measured in eV).

### Synergy From Full Integration of All Components in the Nanocellulose Composite

**FIGURE 4:**
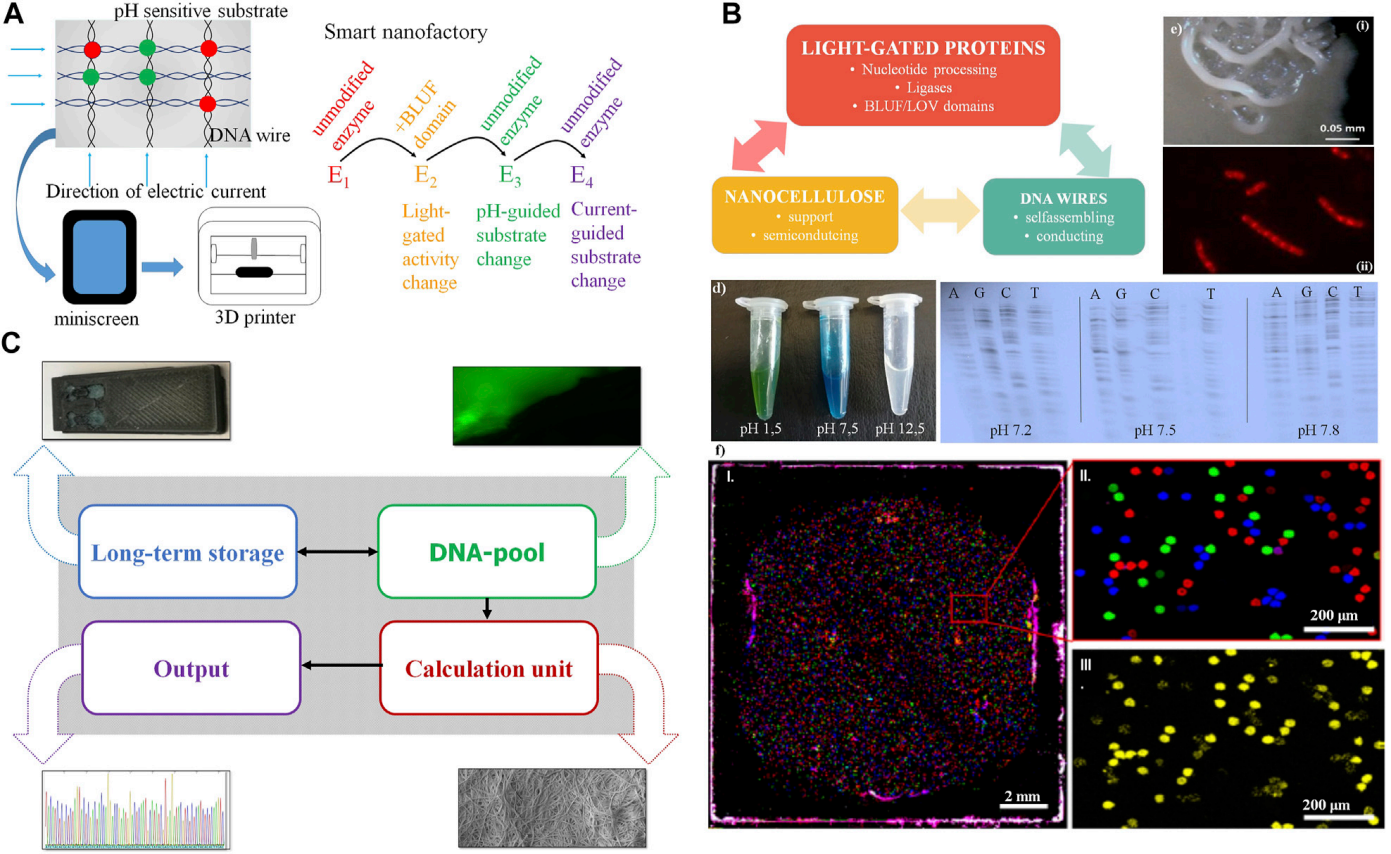
Synergy from full integration of all components in the nanocellulose composite. **(A)** Further nanocellulose composite extensions: Output devices using the nanocellulose chassis as printer or mini screen; light-gated, current- or pH-guided enzymes have accurately controlled activity and substrate specificity by this and complement each other in nanocellulose composite cavities (“nanofactory”) or use the mini wells shown in **(F)**. **(B)** Smart card (or ultimately computer chip) combining nanocellulose, light-gated proteins and DNA wires. **(C)** Demonstrator: we integrate **(i)** DNA for storage (long-term storage); **(ii)** modify the DNA pool by fluorescent nucleotides for read-in or read-out (DNA pool); **(iii)** do efficient calculations combining DNA-processing enzymes, nanocellulose semiconductors, SETs (inset), and classical electronics integrated using the nanocellulose chassis (Jung et al., 2015) (calculation unit), and **(iv)** get efficient sequence read-out (results) by fast sequencing (e.g., next-generation sequencing, output). **(D)** Left: pH-sensitive dye (malachite green) on nanocellulose; right: polymerase (sequenase) specificity are different pH levels. **(E)** Composite biofilm modification: Top: patterning of *B. subtilis* biofilm into spatially defined phenotypic changes by the accumulation of spontaneous *sinR* mutations in a subpopulation of Δ*kinCD* mutant; bottom: cells stained with Nile red. The dye accumulates in damaged regions of the membrane thus membrane-damaged areas are visualized as spotted Nile red areas in bacterial membranes. **(F)** PCR mini wells (Woehrle et al., 2020) with pico-cavities allow specific access to DNA storage molecules. **(i)**: normal size; **(ii)**: enlargement, different DNA molecule species (color); and **(iii)**: specific read-out for one species (yellow).

If all components investigated in Figures 1–3 are integrated into one nanocellulose composite, several synergies arise (summarized in Table 4): This can furthermore be exploited for a 3D printer and nanofactory (Figure 4A) or a performant nanocellulose composite usable as a computer chip (a “CellChip”; Figure 4B).

**TABLE 4:** Optimal performance characteristics.

Individual enzyme	Light-gated polymerase: After switching on this is as fast as normal Klenow polymerase, that is, 40–87 nucleotides per second (Schwartz and Quake, 2009). Using many molecules in parallel is easily possible using micro-wells (Figure 4F). Enzyme signaling can be very fast, 4 nanoseconds after inhibitor binding for tyrosine phosphatases (Chirgadze et al., 2021) and in light-harvesting reaction centers even within picoseconds (Dods et al., 2021). However, to have this much faster speed requires molecular integration of all components as explored in Figure 3. Molecular components we provide for the nanocellulose composite include SETs as well as self-assembling oligos (Saoji and Paukstelis, 2015) and the electronic current-carrying DNA wires (Livshits et al., 2014; Saoji and Paukstelis, 2015; Dvorkin et al., 2018)
Storage	Storage: usage in parallel allows Exabyte (Church et al., 2012); storage of millions of years (Grass et al., 2015); latest actual DNA storage achieved: 250 Terabytes per gram with correction bits; theoretical optimum: 1 Exabyte per kilogram, so 10^18^ bit per kg (Extance, 2016)
Calculation speed	Calculation speed: Use many molecules in parallel (10 million); or fast and direct information processing by light (femtoseconds; references) → Teraflops and very fast processing (up to Petahertz) processing
	Concrete application: Using digital picoliter PCR allows parallelization of up to 1 billion reactions in parallel, each operating on another DNA molecule in a separate well (Wohrle et al., 2020) allowing not only fast library screens but also direct access to different storage items (up to 1 billion) as RAM (random access memory)
	Integration of conventional electronics: gigahertz (Ghz) processors, fast light processing using LED and light-sensitive diodes (Jung et al., 2016).
	Single-electron transistors are biodegradable and have ultralow energy consumption, dimensions are approximately 10 nm (Xu et al., 2020)
Synergy achieves performant devices	(i) Smart DNA storage devices providing fast read-in and out by light-gated enzymes, permanent high-density DNA storage without external energy source and rapid calculations integrating standard electronics;
	(ii) Higher miniaturization exploiting photolithographic techniques would allow full chip integration of nanocellulose SETs and semiconductors, standard electronic components for fast processing, as well as high-density DNA storage, fast read-in and read-out by light-gated processing enzymes, synthesizing and being accessed and modulated by DNA wire networks.
	(iii) Nanofactory or smart (light-gated control) conveyor belt on nanocellulose composite chassis and adapts specificity according to electric current.
	(iv) 3D printer: self-assembling oligos plus DNA wire-grid, at cross pixels directed by pH or voltage selectively opens nanopores for ink (Figure 4H shows the pores we tested).
	(v) Artificial ribosome/translator combining different light-gated enzymes with specific peptide- or nucleotide-specificities.

Table 4 gives more data and resulting optimal performance estimates on 1) individual enzyme performance, 2) high-density storage possibilities for DNA storage, and 3) calculation speed.

A first demonstrator using nanocellulose as chassis is summarized in Figure 4C. Although all major components are available and ready for use and integration, it will take several years of development for full DNA storage performance to be reliably achieved, and full synergy between all components will take even longer. However, for much easier and more reliable output, normal electronics can also be integrated on nanocellulose paper with good performance results (Jung et al., 2015). Moreover, pH change, including pH differences caused by currents, can change the substrate specificity (Figure 4D, left). This is shown for the enzyme sequenase and changed nucleotide specificity for all four nucleotides (Figure 4D, right). Operating DNA storage in this way by fast pH change and incorporating then different substrates from the enzyme according to the applied current is an attractive option. Similarly, either pH change or light activation of polymerase allows, in principle, polymerase activity either bound to the template (copy polymerase) or without the template (*de novo* synthesizing polymerase).

There are numerous further possible refinements of the nanocellulose chassis (Figure 4):

We studied micro-surface patterning (Figure 4Ei) as well as piercing micro-holes (Figure 4Eii) using a *B. subtilis* biofilm with kinase mutations for patterning (see *Materials and Methods*) and compounds such as Nile Red for generation of holes. With similar strategies, one can also fix proteins or other components at specific places of the nanocellulose composite. Another application would be turning the nanocellulose composite into a nanoprinter (Figure 4E, bottom), either to allow the dropping of ink through tiny holes for 3D printing (bacterial membranes of *B. subtilis* stained with Nile Red, the red spots representing cellular damage, in this case, pores) or for connecting active pores (for instance light-gated) between different bacterial membranes or nanocellulose composites.

Finally, we show that we already possess a decent technical alternative to operate our DNA storage by using PCR mini wells (Wöhrle et al., 2020) allowing access to different specific storage contents (Figure 4F).

## Discussion

We show here only proof-of-principle experimental results covering many aspects of a smart nanocellulose composite. Promising for real applications are:

(1) Electronic capabilities including measured semiconductor properties and miniaturization potential shown for the composite in micrographs and *in silico* calculations for a single-electron transistor from nanocellulose.
(2) DNA storage potential includes energy-saving permanent storage with reliable, enzyme-specific controlled read-in and read-out. However, speed is currently still slow while storage density and potential total storage capacity is high (Table 4). Moreover, we show that nanocellulose protects DNA well and has a number of attractive properties as a composite for DNA storage.
(3) Regarding integration of enzymes into nanocellulose composites, this has pre-runners, for example, pH sensors; however, we show here for the first time an active DNA storage, that is, read-in and read-out by light-gated nucleotide-processing enzymes which remain intact and can be used for many cycles.
(4) True molecular integration (Figure 4) shows the highest performance potential, but needs a lot of additional development; even more nanotechnology is required for highest performance, for example, direct molecular integration and synergy. However, with this the potential for a competitor to current electronics would clearly be there, particular regarding energy-saving permanent storage.

### Potential of Nanocellulose Composites

Nanocellulose and nanocellulose composites have several advantageous properties (Pagliaro et al., 2021). Also, classical electronics can easily be integrated (Jung et al., 2015). Similarly, the theoretical advantages of DNA as storage medium have been shown previously including sophisticated storing and encoding schemes (Chen et al., 2020; Chen et al., 2021; Lim et al., 2021). Based on both points, we explore to what extent nanocellulose composites may provide a basis for smart devices: we demonstrate long-lasting DNA storage in nanocellulose, enzymes allow active DNA storage by controlled read-in and writing DNA and read-out or reading DNA using light-gated nucleotide-processing enzymes. Furthermore, the combination of nanocellulose with other substances such as cinnamoyl chloride or copper iodide can provide long-term UV protection of stored DNA, thus preventing its degradation (Zhang et al., 2019; Klochko et al., 2021). Electronic properties of nanocellulose, DNA wires and single-electron transistors (SETs) give the composite even more attractive properties. We show that there are many attractive options for surface structuring of the composite for improved DNA storage: We tested DNA micro-wells and pores, electric and pH-mediated substrate change and pH sensitivity dies. Finally, we examine the strong potential for synergies from all these components.

Light-activating proteins have traditionally been used for optogenetic control and monitoring electrical and biochemical parameters (Paz et al., 2013; Hartsough et al., 2020; Ochoa-Fernandez et al., 2020), but their potential is much higher, for example, we show here that they can be used to control nucleotide activities processing DNA enzymes. As an alternative chemical approach, the recent work of Kesici et al. used two types of photocleavable linkers that were covalently attached to various enzyme types such as polymerase, restriction enzyme and exonuclease for the reversible and controlled activation of proteins (Kesici et al., 2022). Their method is different, using DNA-processing enzymes activated by UV using a specific photocleavable linker. Instead, our approach uses a natural light-gated domain allowing, again and again, activation of the processing enzymes. In our approach, there is also no chemistry involved which means that these light-gated proteins can be synthesized in normal bacteria or eukaryotic cells using the constructs we provide. They even can act inside a cell after activation by light (not investigated here by us). Moreover, the light-gated domains allow specific activation by different wavelengths of different enzymes, so that for instance a general polymerase, a U-specific polymerase, a template-free polymerase or a template-bound (copy) polymerase can each be activated after the other and reused after some time in a new cycle. Each of these constructs (see also Supplementary Table S2 and detailed sequences in Supplementary Material) has been tested in many experiments by us and we compared also different light-gated domains.

The molecular basis how the light-gated protein domains signal and regulate the activity of the subsequent protein has been investigated in several studies. In particular, regarding the BLUF domain molecular details are known (Fujisawa and Masuda, 2018): X-ray crystallography and mutagenesis disclosed that a rearrangement of the hydrogen bond network involving a specific Tyr, Gln, and the FAD cofactor of the BLUF domain are recognized as essential for formation of a BLUF protein signaling state by photoactivation. The hydrogen bond structural change in the active site is propagating in the protein, alters the conformation and transmits the light-induced allosteric signal. Investigating the BLUF domain containing protein PixD from cyanobacterium *Synechocystis* sp. PCC6803, it was found that conformations of the C-terminal helices in the BLUF domain are particularly relevant to signal transduction. The effected intramolecular conformational changes may in some BLUF domain containing proteins even control the interprotein association or dissociation and by this the activity of the protein controlled by the BLUF domain. latest advances in spectroscopy and computation allow now to reveal even more details of the molecular mechanism involved including tackling for example the complex structure of the AppA BLUF protein, which controls photosynthesis and suitable gene expression in the purple bacterium *Rhodobacter sphaeroides* (Hashem et al., 2021).

Nevertheless, Hashem et al. (2021) and Fujisawa and Masuda (2018) are descriptive studies: They nail down specific involved residues of the BLUF domain but do not look at the full protein structure. Moreover, this varies according to the specific protein structure examined. More detail is not known currently according to experimental data.

To get more insight, we did molecular dynamics simulation investigating the BLUF domain (Supplementary Figure S2; Methods described in Supplementary Material): The BLUF–POL interaction hypothesis holds that the photo-activated BLUF domain (its activated conformation) has a higher affinity to the DNA-POL, when it is inactive the affinity decreases. There is a photo-activated BLUF domain available as PBD file (6W72). We show that its conformational energy is high enough to achieve this due to its photoactivation. We used the BLUF domain of BlsA at the ground (green) and photo-activated states (cyan). As you can see, there is a conformational shift of the flexible loop structure upon the BLUF activation (RMSD = 1.76 A) in the residues 110–122 (with a gap: 113 and 114 aa). These residues were predicted as a protein–protein interface to be most likely involved in the interaction with polymerase or T4 polynucleotide kinase. An elevated energy level was also detected starting from −2,185.69 kcal/mol for the ground state to −2,051.68 kcal/mol for the activated state. This mechanism might explain how a BLUF domain interacts with the polymerase or activate it. Similarly, this helps to clarify how a BLUF domain stops or activates the T4 polynucleotide kinase.

The modification potential of our approach is high as demonstrated regarding engineering enzyme specificity for example by detailed engineering of the photo-active enzyme fatty acid photodecarboxylase in its binding pocket, substrate specificity and reaction speed (Amer et al., 2020; Gil et al., 2020). Regarding the control element advocated here, the light-activating domains BLUF or LOV allow for controllable switching on from a few seconds to several tens of minutes (Mathes and Gotze, 2015). Accurate deactivation can be achieved by adding another reporter with a reverse function, such as Opn7b or Dronpa (Karapinar et al., 2021).

Switching the substrate specificity in a controlled way allows writing or reading other nucleotide letters and, even, to change enzyme specificity. Classical but time consuming and irreversible is site-directed mutagenesis. We use such a mutation and achieve a mutated CidI polymerase with a higher preference for adenine. However, a targeted change of substrate and catalytic specificity by electrical current or pH as shown here for the DNA polymerase sequenase is particularly promising as reversible, fast and applicable to any enzyme of choice. Similarly, DNA can be switched, for instance, a pH modifying dye was used to achieve a light-driven conformational switch in i-motif DNA (Liu et al., 2007). This allows use of nucleotide-processing enzymes in several modes, for example, as template-bound copy polymerases (Klenow polymerase, sequenase) and, after a switch by current, pH or light as template-free polymerase to incorporate the nucleotide of choice. We stress that we show here only proof-of-principle for all these nucleotide-processing enzyme modifications. Accurate and fast performance will require a lot of developmental work.

### Nanocellulose Composite Electronic Properties

Nanocellulose can be rendered conducting by the addition of metal ions (Silva et al., 2020) or graphene (Yang et al., 2017). We show here that it can be rendered semi-conductive by iodine doping at different concentrations. Moreover, we show the fine structure of nanocellulose allows using miniature gates and junctions, which presents in principle an attractive alternative to silicon. To further explore this, we present simulation data on how a SET device made of nanocellulose would work, including molecular orbital calculations. By decreasing the gap between the orbitals we can increase the conductivity and vice versa. Indeed, the energy gap between HOMO (highest occupied molecular orbital) and LUMO (lowest unoccupied molecular orbital) determines if the material is a conductor, insulator or semiconductor. Therefore, if the energy gap is small (e.g., in metals) the electrons can jump easily from the HOMO to LUMO orbitals thus the material is a conductor. If the energy gap is very high, the material is an insulator and if it is between conductor and insulator, the material is a semiconductor (useful for a transistor). In our case, the nanocellulose is probably a weak insulator (or not very good semiconductor) *per se* and it becomes the semiconductor after a doping process, which was precisely modeled *in silico* using DFT calculations on a SET device using well established techniques (Shityakov et al., 2017) (Table 3). As a sensitive molecular transistor, we show this here for the first time and stress the high potential of nanocellulose in this respect. However, photolithographic techniques for nano-structuring nanocellulose required for such devices to get appropriate connections for the transistor device were not attempted.

The electronic properties in nanocellulose are a new and interesting observation. This was confirmed by us by repeated measurements (at least triplicates). Moreover, Jung et al. (2015) showed that nanocellulose is a well-suited host material for conventional electronic parts. We show now as a new property that nanocellulose itself can be titrated from full conducting to semiconducting and not conducting, this indicates by the experimental data here a new area for applications of nanocellulose.

We found that the fine structure of the nanocellulose shows tiny bridges and bifurcations and that these remain also after iodine treatment (Figure 3B). However, to target these structures is quite a challenge: to enable tiny conducting wires transporting current on the gate and then measure the current through the other two contacts as required for a transistor is a major project needing years in a fine-structure lab. So instead, we simulated this only *in silico* including detailed molecular calculations. The theoretical calculation looked at the properties a single-electron transistor made from nanocellulose would have. They confirm the electronic properties we found in the experiment looking after the iodine doping. The detailed molecular structure of the SET is also shown (Figure 3E). We show by calculation of HOMO LUMO orbitals and gap energy that such a transistor made from nanocellulose is calculated to work efficiently as a SET. The results are comparable to those for two other SETs, an Indigo and a Tyrian Purple Single-Electron Nano-Transistor, respectively (Shityakov et al., 2017). Hence, the potential for powerful electronic capabilities by nanocellulose is there, though full experimental proof of transistor properties is beyond our current capabilities, instead we just calculate the properties of such a device and show that nanocellulose has a suitable fine structure to allow such usage.

### Nanocellulose Smart Card Synergies

The combination of a nanocellulose chassis, nucleotide-processing enzymes and electronic properties is a major new contribution with powerful synergies (Figure 4; Table 4). For instance, we can use DNA wires on the nanocellulose and change the DNA wires by synthesis from the light-controlled nucleotide-processing enzymes. Similarly, the current transported by the DNA wires can be used to change the substrate specificity of the nucleotide-processing enzymes. Moreover, for rapid calculations nanocellulose is an optimal chassis for classical electronic parts. Furthermore, LEDs can be used for efficient operation of the nucleotide-processing enzymes of the DNA storage while the computing is done by the latest silicon chips. Our nanocellulose composite was further modified by applying nanotechnologies such as micro-pores, nanopatterning and picoliter DNA assay wells. Table 4 illustrates the large potential of the individual components as well as some of the synergies possible.

Regarding optimized production of a nanocellulose DNA storage chip device, there is an efficient scale-up possible regarding energy and speed using digital PCR (Wöhrle et al., 2020). To obtain a functional device one could use 3D printing combined with standard photolithographic techniques to generate computer chips. We are currently testing our constructs in such a 3D printer setting. Together, this illustrates that there is already now a good potential for this approach for miniaturization and efficient printing and several working methods are at hand. However, there is still a considerable road to go regarding development and optimization to achieve efficient and cheap high-throughput production.

## Conclusion

In this article, we show the huge potential of nanocellulose composites for information storage and smart card devices. While DNA-based long-term high-density storage capabilities are well known and undisputed (Extance, 2016), we demonstrate here the potential to tackle open challenges. Encompassed in such a device including DNA storage read-in and read-out using light-gated enzymes, it is possible to achieve fast processing by using light. This has very high potential, a proof of concept was explored but there is still a long road to get high performance. Electronic capabilities of nanocellulose are demonstrated by us including miniaturization and applying DNA wires (available, tested by us in place). In addition, direct integration of electronic components as well as many nanotechnology techniques improve this, such as pH-sensitive colors, integrating patterned bacterial membranes, nanopores, micro-wells, and digital PCR. This allows steady refinement of the nanocellulose composite. Hence, we are confident that the nanocellulose chip is an attractive device with high potential. However, for competitive applications compared to typical electronic devices, longer development is still needed. It will then achieve a cheap, environmentally friendly long-term DNA storage. Future fast processing capacity will profit from direct integration of classical electronic components (transistors, LEDs) with novel components explored here such as light-gated nucleotide-processing enzymes, semiconducting nanocellulose with SETs, DNA wires, and oligonucleotides.

## Data Availability

The original contributions presented in the study are included in the article/Supplementary Material; further inquiries can be directed to the corresponding author.
