# Supplementary material for: Nanocellulose Composites as Smart Devices With Chassis, Light-Directed DNA Storage, Engineered Electronic Properties, and Chip Integration
Source: Front Bioeng Biotechnol. 2022 Aug 8;10:869111. doi: 10.3389/fbioe.2022.869111 (PMC9465592; doi:10.3389/fbioe.2022.869111)
Supplement: Supplementary file 1 [file DataSheet1.docx]

Supplementary Material

This file contains

**- all Supplementary Data** (part I: structural analysis of BLUF domain, part II: sequences of the constructs used),

- Supplementary Figure 1: DNA storage sequencing

- Supplementary Figure 2: Comparing the BLUF domain of BlsA at the ground (green) and photo-activated states (cyan). (with supplementary methods)

**- all Supplementary Tables** (List of available light-gated protein constructs used in our investigation, list of oligonucleotides used in our investigation)

# Supplementary Data

**Part I. Structural analysis of the BLUF domain**

The BlsA domain of BLUF domain was examined at the ground (green) and photo-activated states (cyan) (Supplementary figure 2). We found a conformational shift of the flexible loop structure upon the BLUF activation (RMSD = 1.76 A) within the residues 110-122 (with a gap: 113 and 114 aa). These residues were predicted as a protein-protein interface to be most likely involved in the interaction with polymerase or T4 polynucleotide kinase. An elevated Epot level was detected starting from -2185.69 kcal/mol for the ground state to -2051.68 kcal/mol for the activated state. This mechanism might explain how a BLUF domain interacts with the polymerase or activate it. Similarly, this helps to clarify how a BLUF domain stops or activates the T4 polynucleotide kinase.

Methods

The ground and photo-activated states of the BlsA BLUF domain were obtained from the Protein Data Bank with the corresponding accession numbers (6W6Z and 6W72) as the X-ray diffraction models (Chitrakar et al., 2020). The protein structures were refined using the user template homology modelling integrated into the SWISS-MODEL pipeline (Kopp and Schwede, 2006). The RMSD (root-mean-square deviation) and potential energy (Epot) values were measured using the PyMOL in-house scripts and the Molecular Operating Environment software (Shityakov et al., 2021; Chemical Computing Group Inc., Montreal, QC, Canada). The consensus protein-protein interaction (PPI) site predictor was applied to predict the PPI amino acid residues for the BLUF domain of BlsA (Zhou and Shan, 2001).

References:

Chitrakar, I., et al. (2020). "Structural Basis for the Regulation of Biofilm Formation and Iron Uptake in A. baumannii by the Blue-Light-Using Photoreceptor, BlsA." ACS Infect Dis 6(10): 2592-2603.

Kopp, J. and T. Schwede (2006). "The SWISS-MODEL Repository: new features and functionalities." Nucleic Acids Res 34(Database issue): D315-318.

Shityakov, S., et al. (2021). "Scaffold Searching of FDA and EMA-Approved Drugs Identifies Lead Candidates for Drug Repurposing in Alzheimer's Disease." Front Chem 9: 736509.

Zhou, H. X. and Y. Shan (2001). "Prediction of protein interaction sites from sequence profile and residue neighbor list." Proteins 44(3): 336-343.

**Part II. Sequences of the enzymes:**

Blue – BLUF-sequence. Orange – linker. Black – enzyme sequence. Italics – restriction site

GFP

*AGATCT*ATGCTTACCACCCTTATTTATCGTAGCCATATACGTGACGACGAACCTGTCAAAAAAATCGAAGAAATGGTTTCGATAGCAAATCGCAGGAACATGCAGTCTGACGTAACAGGGATCTTACTGTTTAATGGTTCTCATTTTTTCCAGCTTCTGGAAGGTCCGGAAGAACAGGTTAAAATGATATATCGGGCTATATGCCAGGATCCACGGCACTATAATATTGTTGAGCTGCTGTGCGATTACGCGCCTGCTCGCCGTTTTGGCAAAGCGGGAATGGAATTATTTGATTTGCGCCTGCACGAGCGAGATGACGTTTTACAGGCCGTATTCGACAAAGGCACATCAAAATTTCAGCTAACTTATGATGACAGAGCG*AAGCTT*

BLUF-Exonuclease

ATGCTTACCACCCTTATTTATCGTAGCCATATACGTGACGACGAACCTGTCAAAAAAATCGAAGAAATGGTTTCGATAGCAAATCGCAGGAACATGCAGTCTGACGTAACAGGGATCTTACTGTTTAATGGTTCTCATTTTTTCCAGCTTCTGGAAGGTCCGGAAGAACAGGTTAAAATGATATATCGGGCTATATGCCAGGACCCACGGCACTATAATATTGTTGAGCTGCTGTGCGATTACGCGCCTGCTCGCCGTTTTGGCAAAGCGGGAATGGAATTATTTGATTTGCGCCTGCACGAGCGAGATGACGTTTTACAGGCCGTATTCGACAAAGGCACATCAAAATTTCAGCTAACTTATGATGACAGAGCGGGGAGCATGATGAATGACGGTAAGCAACAATCTACCTTTTTGTTTCACGATTACGAAACCTTTGGCACGCACCCCGCGTTAGATCGCCCTGCACAGTTCGCAGCCATTCGCACCGATAGCGAATTCAATGTCATCGGCGAACCCGAAGTCTTTTACTGCAAGCCCGCTGATGACTATTTACCCCAGCCAGGAGCCGTATTAATTACCGGTATTACCCCGCAGGAAGCACGGGCGAAAGGAGAAAACGAAGCCGCGTTTGCCGCCCGTATTCACTCGCTTTTTACCGTACCGAAGACCTGTATTCTGGGCTACAACAATGTGCGTTTCGACGACGAAGTCACACGCAACATTTTTTATCGTAATTTCTACGATCCTTACGCCTGGAGCTGGCAGCATGATAACTCGCGCTGGGATTTACTGGATGTTATGCGTGCCTGTTATGCCCTGCGCCCGGAAGGAATAAACTGGCCTGAAAATGATGACGGTCTACCGAGCTTTCGCCTTGAGCATTTAACCAAAGCGAATGGTATTGAACATAGCAACGCCCACGATGCGATGGCTGATGTGTACGCCACTATTGCGATGGCAAAGCTGGTAAAAACGCGTCAGCCACGCCTGTTTGATTATCTCTTTACCCATCGTAATAAACACAAACTGATGGCGTTGATTGATGTTCCGCAGATGAAACCCCTGGTGCACGTTTCCGGAATGTTTGGAGCATGGCGCGGCAATACCAGCTGGGTGGCACCGCTGGCGTGGCATCCTGAAAATCGCAATGCCGTAATTATGGTGGATTTGGCAGGAGACATTTCGCCATTACTGGAACTGGATAGCGACACATTGCGCGAGCGTTTATATACCGCAAAAACCGATCTTGGCGATAACGCCGCCGTTCCGGTTAAGCTGGTGCATATCAATAAATGTCCGGTGCTGGCCCAGGCGAATACGCTACGCCCGGAAGATGCCGACCGACTGGGAATTAATCGTCAGCATTGCCTCGATAACCTGAAAATTCTGCGTGAAAATCCGCAAGTGCGCGAAAAAGTGGTGGCGATATTCGCGGAAGCCGAACCGTTTACGCCTTCAGATAACGTGGATGCACAGCTTTATAACGGCTTTTTCAGTGACGCAGATCGTGCAGCAATGAAAATTGTGCTGGAAACCGAGCCGCGTAATTTACCGGCACTGGATATCACTTTTGTTGATAAACGGATTGAAAAGCTGTTGTTCAATTATCGGGCACGCAACTTCCCGGGGACGCTGGATTATGCCGAGCAGCAACGCTGGCTGGAGCACCGTCGCCAGGTCTTCACGCCAGAGTTTTTGCAGGGTTATGCTGATGAATTGCAGATGCTGGTACAACAATATGCCGATGACAAAGAGAAAGTGGCGCTGTTAAAAGCACTTTGGCAGTACGCGGAAGAGATTGTC

BLUF-T4 kinase

ATGCTTACCACCCTTATTTATCGTAGCCATATACGTGACGACGAACCTGTCAAAAAAATCGAAGAAATGGTTTCGATAGCAAATCGCAGGAACATGCAGTCTGACGTAACAGGGATCTTACTGTTTAATGGTTCTCATTTTTTCCAGCTTCTGGAAGGTCCGGAAGAACAGGTTAAAATGATATATCGGGCTATATGCCAGGACCCACGGCACTATAATATTGTTGAGCTGCTGTGCGATTACGCGCCTGCTCGCCGTTTTGGCAAAGCGGGAATGGAATTATTTGATTTGCGCCTGCACGAGCGAGATGACGTTTTACAGGCCGTATTCGACAAAGGCACATCAAAATTTCAGCTAACTTATGATGACAGAGCGGGGAGCATGAAAAAGATTATTTTGACTATTGGCTGTCCTGGTTCTGGTAAGAGTACTTGGGCTCGTGAATTTATTGCTAAGAATCCCGGGTTTTATAATATCAATCGTGATGACTATCGCCAATCTATTATGGCGCATGAAGAACGCGATGAGTACAAGTATACCAAAAAGAAAGAAGGTATCGTAACTGGTATGCAGTTTGATACAGCTAAAAGTATTCTGTACGGTGGCGATTCTGTTAAGGGAGTAATCATTTCAGATACTAACCTGAATCCTGAACGTCGCCTAGCATGGGAAACTTTTGCCAAAGAATACGGCTGGAAAGTTGAACATAAAGTGTTTGATGTTCCTTGGACTGAATTGGTTAAACGTAACTCAAAACGCGGAACTAAAGCAGTACCAATTGATGTTTTACGTTCAATGTATAAAAGCATGCGAGAGTATCTCGGTCTTCCAGTATATAATGGGACTCCTGGTAAACCAAAAGCAGTTATTTTTGATGTTGATGGTACACTAGCTAAAATGAATGGTCGTGGTCCTTATGACCTTGAAAAATGCGATACCGATGTTATCAATCCTATGGTTGTTGAACTGTCTAAGATGTATGCTCTTATGGGTTATCAAATCGTAGTCGTTTCAGGTCGTGAAAGTGGAACTAAAGAAGACCCAACGAAATATTATCGTATGACCCGTAAATGGGTTGAGGACATTGCTGGCGTTCCATTAGTTATGCAATGTCAGCGCGAACAAGGCGATACCCGTAAAGACGATGTAGTTAAAGAAGAAATTTTCTGGAAACACATTGCACCGCATTTTGACGTGAAATTAGCTATTGATGACCGAACTCAAGTAGTTGAAATGTGGCGTCGTATCGGTGTTGAATGCTGGCAAGTCGCTTCGGGAGATTTT

BLUF-Cid1 polymerase (short version)

CTGGTCCCGCGTGGCTCGCTGACGACGCTGATTTACCGTTCACACATCCGTGACGACGAACCGGTCAAGAAAATTGAAGAAATGGTGTCCATCGCAAACCGTCGCAATATGCAGTCAGATGTTACCGGCATTCTGCTGTTTAATGGTAGCCATTTCTTTCAGCTGCTGGAAGGCCCGGAAGAACAAGTGAAAATGATTTATCGTGCGATCTGCCAAGATCCGCGCCACTATAACATCGTTGAACTGCTGTGTGACTACGCACCGGCTGCGCGCGGCGGTGGCGGATCCGGAGGCGGCGGTTCCGGAGGCGGCGGATCCGCGCGCTCCTATCAGAAAGTTCCGAACAGTCACAAAGAATTCACGAAATTCTGTTACGAAGTCTACAACGAAATCAAAATCTCCGATAAAGAATTCAAAGAAAAACGTGCAGCTCTGGACACCCTGCGTCTGTGCCTGAAACGCATTTCACCGGATGCGGAACTGGTGGCCTTTGGCAGCCTGGAATCTGGTCTGGCGCTGAAAAATTCGGATATGGACCTGTGTGTTCTGATGGATAGCCGCGTCCAGTCTGACACGATTGCACTGCAATTTTATGAAGAACTGATCGCTGAAGGCTTTGAGGGTAAATTCCTGCAGCGTGCCCGCATTCCGATTATCAAACTGACCTCGGATACGAAAAATGGCTTTGGTGCGAGCTTCCAATGTGACATTGGCTTTAACAATCGCCTGGCCATCCATAACACGCTGCTGCTGAGTTCCTATACCAAACTGGATGCACGTCTGAAACCGATGGTTCTGCTGGTCAAACACTGGGCTAAACGCAAACAGATTAATTCTCCGTACTTCGGCACCCTGTCATCGTATGGTTACGTGCTGATGGTTCTGTATTACCTGATTCATGTCATCAAACCGCCGGTGTTTCCGAACCTGCTGCTGAGTCCGCTGAAACAGGAAAAAATTGTTGATGGCTTTGACGTCGGTTTCGATGACAAACTGGAAGATATCCCGCCGTCTCAAAATTATAGCTCTCTGGGCAGTCTGCTGCACGGCTTTTTCCGTTTCTACGCATACAAATTCGAACCGCGCGAAAAAGTGGTTACGTTCCGTCGCCCGGATGGCTACCTGACCAAACAGGAAAAAGGTTGGACCAGCGCAACGGAACATACCGGCTCAGCTGATCAAATCATCAAAGACCGTTACATCCTGGCCATCGAAGATCCGTTTGAAATTAGCCACAATGTTGGTCGCACCGTCAGTTCCTCAGGCCTGTACCGTATCCGTGGTGAATTCATGGCGGCCTCACGTCTGCTGAACTCACGCTCGTATCCGATTCCGTACGATTCGCTGTTTGAAGAAGCGCCGATCCCGCCGCGTCGCCAGAAAAAGACCGATGAACAATCTAATAAAAAACTGCTGAATGAAACCGATGGCGATAACTCCGAACTGGTCCCGCGTGGCTCG

BLUF-Cid1 polymerase (long version/full BLUF)

CTGGTCCCGCGTGGCTCGCTGACGACGCTGATTTACCGTTCACACATCGGTGACGACGAAGCGGTCAAGAAAATTGAAAAAATGGGTGTCCCAGTCGACACATACGCGGCTGCTGACAATACTAATTGGGGCTACGAGTTCAAGGCATTGGGTTTTTTCCGCACGTTTGTGCTGGCCACGGAACAGAGCACCTACTTCGAAATTCCGGCGGAAGATTCGTGGCTGTTTATCGCCGATGGTAGCGACAAAGAACTGGACAGTTGCGCACTGTCCCCGACCATTAATGATCATTTTGCTTTCCACCCGATCGTTGACCCGCTGAGCCGTCGCATTATCGCGTTCGAAGCCATTGTCCAGAAAAACGAAGATTCGCCGAGCGCGATTGCCGTGGGCCAACGTAAAGATGGTGAAATCTATACGGCCGACCTGAAATCTAAAGCACTGGCTTTTACCATGGCGCATGCCCTGGAACTGGGTGATAAAATGATTAGCATCAACCTGCTGCCGATGACGCTGGTGAATGAACCGGACGCAGTTTCTTTCCTGCTGAACGAAATTAAAGCAAATGCTCTGGTCCCGGAACAGATTATCGTGGAATTTACCGAATCAGAAGTGATTTCGCGTTTTGATGAATTCGCGGAAGCCATCAAATCTCTGAAAGCGGCCGGCATTAGTGTTGCGATCGATCATTTTGGCGCAGGTTTCGCTGGTCTGCTGCTGCTGTCTCGTTTTCAGCCGGACCGCATTAAAATCAGTCAAGAACTGATTACCAACGTGCACAAATCCGGCCCGCGCCAGGCGATTATCCAAGCCATTATCAAATGCTGTACGTCGCTGGAAATTCAAGTGAGCGCAATGGGTGTTGCTACCCCGGAAGAATGGATGTGGCTGGAATCAGCGGGCATCGAAATGTTTCAAGGTGACCTGTTTGCAAAAGCTAAACTGAACGGCATTCCGTCCATCGCATGGCCGGAGAAAAAAGCGCGCGGCGGTGGCGGATCCGGAGGCGGCGGTTCCGGAGGCGGCGGATCCGCGCGCTCCTATCAGAAAGTTCCGAACAGTCACAAAGAATTCACGAAATTCTGTTACGAAGTCTACAACGAAATCAAAATCTCCGATAAAGAATTCAAAGAAAAACGTGCAGCTCTGGACACCCTGCGTCTGTGCCTGAAACGCATTTCACCGGATGCGGAACTGGTGGCCTTTGGCAGCCTGGAATCTGGTCTGGCGCTGAAAAATTCGGATATGGACCTGTGTGTTCTGATGGATAGCCGCGTCCAGTCTGACACGATTGCACTGCAATTTTATGAAGAACTGATCGCTGAAGGCTTTGAGGGTAAATTCCTGCAGCGTGCCCGCATTCCGATTATCAAACTGACCTCGGATACGAAAAATGGCTTTGGTGCGAGCTTCCAATGTGACATTGGCTTTAACAATCGCCTGGCCATCCATAACACGCTGCTGCTGAGTTCCTATACCAAACTGGATGCACGTCTGAAACCGATGGTTCTGCTGGTCAAACACTGGGCTAAACGCAAACAGATTAATTCTCCGTACTTCGGCACCCTGTCATCGTATGGTTACGTGCTGATGGTTCTGTATTACCTGATTCATGTCATCAAACCGCCGGTGTTTCCGAACCTGCTGCTGAGTCCGCTGAAACAGGAAAAAATTGTTGATGGCTTTGACGTCGGTTTCGATGACAAACTGGAAGATATCCCGCCGTCTCAAAATTATAGCTCTCTGGGCAGTCTGCTGCACGGCTTTTTCCGTTTCTACGCATACAAATTCGAACCGCGCGAAAAAGTGGTTACGTTCCGTCGCCCGGATGGCTACCTGACCAAACAGGAAAAAGGTTGGACCAGCGCAACGGAACATACCGGCTCAGCTGATCAAATCATCAAAGACCGTTACATCCTGGCCATCGAAGATCCGTTTGAAATTAGCCACAATGTTGGTCGCACCGTCAGTTCCTCAGGCCTGTACCGTATCCGTGGTGAATTCATGGCGGCCTCACGTCTGCTGAACTCACGCTCGTATCCGATTCCGTACGATTCGCTGTTTGAAGAAGCGCCGATCCCGCCGCGTCGCCAGAAAAAGACCGATGAACAATCTAATAAAAAACTGCTGAATGAAACCGATGGCGATAACTCCGAACTGGTCCCGCGTGGCTCG

BLUF-Cid1 polymerase (long version/mutation H336D)

CTGGTCCCGCGTGGCTCGCTGACGACGCTGATTTACCGTTCACACATCGGTGACGACGAAGCGGTCAAGAAAATTGAAAAAATGGGTGTCCCAGTCGACACATACGCGGCTGCTGACAATACTAATTGGGGCTACGAGTTCAAGGCATTGGGTTTTTTCCGCACGTTTGTGCTGGCCACGGAACAGAGCACCTACTTCGAAATTCCGGCGGAAGATTCGTGGCTGTTTATCGCCGATGGTAGCGACAAAGAACTGGACAGTTGCGCACTGTCCCCGACCATTAATGATCATTTTGCTTTCCACCCGATCGTTGACCCGCTGAGCCGTCGCATTATCGCGTTCGAAGCCATTGTCCAGAAAAACGAAGATTCGCCGAGCGCGATTGCCGTGGGCCAACGTAAAGATGGTGAAATCTATACGGCCGACCTGAAATCTAAAGCACTGGCTTTTACCATGGCGCATGCCCTGGAACTGGGTGATAAAATGATTAGCATCAACCTGCTGCCGATGACGCTGGTGAATGAACCGGACGCAGTTTCTTTCCTGCTGAACGAAATTAAAGCAAATGCTCTGGTCCCGGAACAGATTATCGTGGAATTTACCGAATCAGAAGTGATTTCGCGTTTTGATGAATTCGCGGAAGCCATCAAATCTCTGAAAGCGGCCGGCATTAGTGTTGCGATCGATCATTTTGGCGCAGGTTTCGCTGGTCTGCTGCTGCTGTCTCGTTTTCAGCCGGACCGCATTAAAATCAGTCAAGAACTGATTACCAACGTGCACAAATCCGGCCCGCGCCAGGCGATTATCCAAGCCATTATCAAATGCTGTACGTCGCTGGAAATTCAAGTGAGCGCAATGGGTGTTGCTACCCCGGAAGAATGGATGTGGCTGGAATCAGCGGGCATCGAAATGTTTCAAGGTGACCTGTTTGCAAAAGCTAAACTGAACGGCATTCCGTCCATCGCATGGCCGGAGAAAAAAGCGCGCGGCGGTGGCGGATCCGGAGGCGGCGGTTCCGGAGGCGGCGGATCCGCGCGCTCCTATCAGAAAGTTCCGAACAGTCACAAAGAATTCACGAAATTCTGTTACGAAGTCTACAACGAAATCAAAATCTCCGATAAAGAATTCAAAGAAAAACGTGCAGCTCTGGACACCCTGCGTCTGTGCCTGAAACGCATTTCACCGGATGCGGAACTGGTGGCCTTTGGCAGCCTGGAATCTGGTCTGGCGCTGAAAAATTCGGATATGGACCTGTGTGTTCTGATGGATAGCCGCGTCCAGTCTGACACGATTGCACTGCAATTTTATGAAGAACTGATCGCTGAAGGCTTTGAGGGTAAATTCCTGCAGCGTGCCCGCATTCCGATTATCAAACTGACCTCGGATACGAAAAATGGCTTTGGTGCGAGCTTCCAATGTGACATTGGCTTTAACAATCGCCTGGCCATCCATAACACGCTGCTGCTGAGTTCCTATACCAAACTGGATGCACGTCTGAAACCGATGGTTCTGCTGGTCAAACACTGGGCTAAACGCAAACAGATTAATTCTCCGTACTTCGGCACCCTGTCATCGTATGGTTACGTGCTGATGGTTCTGTATTACCTGATTCATGTCATCAAACCGCCGGTGTTTCCGAACCTGCTGCTGAGTCCGCTGAAACAGGAAAAAATTGTTGATGGCTTTGACGTCGGTTTCGATGACAAACTGGAAGATATCCCGCCGTCTCAAAATTATAGCTCTCTGGGCAGTCTGCTGCACGGCTTTTTCCGTTTCTACGCATACAAATTCGAACCGCGCGAAAAAGTGGTTACGTTCCGTCGCCCGGATGGCTACCTGACCAAACAGGAAAAAGGTTGGACCAGCGCAACGGAACATACCGGCTCAGCTGATCAAATCATCAAAGACCGTTACATCCTGGCCATCGAAGATCCGTTTGAAATTAGCGACAATGTTGGTCGCACCGTCAGTTCCTCAGGCCTGTACCGTATCCGTGGTGAATTCATGGCGGCCTCACGTCTGCTGAACTCACGCTCGTATCCGATTCCGTACGATTCGCTGTTTGAAGAAGCGCCGATCCCGCCGCGTCGCCAGAAAAAGACCGATGAACAATCTAATAAAAAACTGCTGAATGAAACCGATGGCGATAACTCCGAACTGGTCCCGCGTGGCTCG

BLUF-Cid1 polymerase (long version/mutation H336A)

CTGGTCCCGCGTGGCTCGCTGACGACGCTGATTTACCGTTCACACATCGGTGACGACGAAGCGGTCAAGAAAATTGAAAAAATGGGTGTCCCAGTCGACACATACGCGGCTGCTGACAATACTAATTGGGGCTACGAGTTCAAGGCATTGGGTTTTTTCCGCACGTTTGTGCTGGCCACGGAACAGAGCACCTACTTCGAAATTCCGGCGGAAGATTCGTGGCTGTTTATCGCCGATGGTAGCGACAAAGAACTGGACAGTTGCGCACTGTCCCCGACCATTAATGATCATTTTGCTTTCCACCCGATCGTTGACCCGCTGAGCCGTCGCATTATCGCGTTCGAAGCCATTGTCCAGAAAAACGAAGATTCGCCGAGCGCGATTGCCGTGGGCCAACGTAAAGATGGTGAAATCTATACGGCCGACCTGAAATCTAAAGCACTGGCTTTTACCATGGCGCATGCCCTGGAACTGGGTGATAAAATGATTAGCATCAACCTGCTGCCGATGACGCTGGTGAATGAACCGGACGCAGTTTCTTTCCTGCTGAACGAAATTAAAGCAAATGCTCTGGTCCCGGAACAGATTATCGTGGAATTTACCGAATCAGAAGTGATTTCGCGTTTTGATGAATTCGCGGAAGCCATCAAATCTCTGAAAGCGGCCGGCATTAGTGTTGCGATCGATCATTTTGGCGCAGGTTTCGCTGGTCTGCTGCTGCTGTCTCGTTTTCAGCCGGACCGCATTAAAATCAGTCAAGAACTGATTACCAACGTGCACAAATCCGGCCCGCGCCAGGCGATTATCCAAGCCATTATCAAATGCTGTACGTCGCTGGAAATTCAAGTGAGCGCAATGGGTGTTGCTACCCCGGAAGAATGGATGTGGCTGGAATCAGCGGGCATCGAAATGTTTCAAGGTGACCTGTTTGCAAAAGCTAAACTGAACGGCATTCCGTCCATCGCATGGCCGGAGAAAAAAGCGCGCGGCGGTGGCGGATCCGGAGGCGGCGGTTCCGGAGGCGGCGGATCCGCGCGCTCCTATCAGAAAGTTCCGAACAGTCACAAAGAATTCACGAAATTCTGTTACGAAGTCTACAACGAAATCAAAATCTCCGATAAAGAATTCAAAGAAAAACGTGCAGCTCTGGACACCCTGCGTCTGTGCCTGAAACGCATTTCACCGGATGCGGAACTGGTGGCCTTTGGCAGCCTGGAATCTGGTCTGGCGCTGAAAAATTCGGATATGGACCTGTGTGTTCTGATGGATAGCCGCGTCCAGTCTGACACGATTGCACTGCAATTTTATGAAGAACTGATCGCTGAAGGCTTTGAGGGTAAATTCCTGCAGCGTGCCCGCATTCCGATTATCAAACTGACCTCGGATACGAAAAATGGCTTTGGTGCGAGCTTCCAATGTGACATTGGCTTTAACAATCGCCTGGCCATCCATAACACGCTGCTGCTGAGTTCCTATACCAAACTGGATGCACGTCTGAAACCGATGGTTCTGCTGGTCAAACACTGGGCTAAACGCAAACAGATTAATTCTCCGTACTTCGGCACCCTGTCATCGTATGGTTACGTGCTGATGGTTCTGTATTACCTGATTCATGTCATCAAACCGCCGGTGTTTCCGAACCTGCTGCTGAGTCCGCTGAAACAGGAAAAAATTGTTGATGGCTTTGACGTCGGTTTCGATGACAAACTGGAAGATATCCCGCCGTCTCAAAATTATAGCTCTCTGGGCAGTCTGCTGCACGGCTTTTTCCGTTTCTACGCATACAAATTCGAACCGCGCGAAAAAGTGGTTACGTTCCGTCGCCCGGATGGCTACCTGACCAAACAGGAAAAAGGTTGGACCAGCGCAACGGAACATACCGGCTCAGCTGATCAAATCATCAAAGACCGTTACATCCTGGCCATCGAAGATCCGTTTGAAATTAGCGCGAATGTTGGTCGCACCGTCAGTTCCTCAGGCCTGTACCGTATCCGTGGTGAATTCATGGCGGCCTCACGTCTGCTGAACTCACGCTCGTATCCGATTCCGTACGATTCGCTGTTTGAAGAAGCGCCGATCCCGCCGCGTCGCCAGAAAAAGACCGATGAACAATCTAATAAAAAACTGCTGAATGAAACCGATGGCGATAACTCCGAACTGGTCCCGCGTGGCTCG

BLUF-Adenyltransferase

ATGCTTACCACCCTTATTTATCGTAGCCATATACGTGACGACGAACCTGTCAAAAAAATCGAAGAAATGGTTTCGATAGCAAATCGCAGGAACATGCAGTCTGACGTAACAGGGATCTTACTGTTTAATGGTTCTCATTTTTTCCAGCTTCTGGAAGGTCCGGAAGAACAGGTTAAAATGATATATCGGGCTATATGCCAGGACCCACGGCACTATAATATTGTTGAGCTGCTGTGCGATTACGCGCCTGCTCGCCGTTTTGGCAAAGCGGGAATGGAATTATTTGATTTGCGCCTGCACGAGCGAGATGACGTTTTACAGGCCGTATTCGACAAAGGCACATCAAAATTTCAGCTAACTTATGATGACAGAGCGGGGAGCATGGGTGAATTCTTTCCTGCACAAATTTCCGAGCAGCTATCCCACGCTCGCGGGGTGATCGAGCGCCATCTGGCTGCAACGCTGGACACAATCCACCTGTTCGGATCTGCGCTCGATGGAGGGTTGAAGCCGGACAGCGACATCGACTTGCTCGTGACCGTCAGCGCCGCACCTAACGATTCGCTCCGGCAGGCACTAATGCTCGACCTGCTAAAAGTCTCATCACCGCCAGGCGATGGCGGACCATGGCGACCGCTGGAGGTGACTGTTGTCGCTCGAAGCGAAGTAGTGCCCTGGCGCTATCCGGCGATACGTGAGCTTCAGTTCGGTGAGTGGCTCCGCCACGACATCCTCTCCGGAACGTTCGAGCCTTCCGTTCTGGATCACGATCTTGCGATTTTGCTGACCAAGGCGAGGCAACACAGCCTTGCTCTGCTAGGTCCATCCGCAGTCACGTTCTTCGAGCCGGTGCCGAACGAGCATTTTTCCAAGGCGCTTTTCGACACGATTGCCCAGTGGAATTCAGAGTCGGATTGGAAGGGTGACGAGCGGAACGTCGTTCTTGCTCTTGCTCGCATTTGGTACAGTGCTTCAACGGGTCTCATTGCTCCTAAGGACGTTGCTGCCGCATGGGTATCGGAGCGTTTGCCTGCCGAGCATCGGCCCATCATTTGCAAGGCACGCGCGGCGTACCTGGGTAGCGAGGACGACGACCTAGCAATGCGCGTCGAAGAGACGGCTGCGTTCGTTCGATATGCCAAAGCAACGATTGAGAGAATCTTGCGT

LOV-Taq polymerase

TTGGCTACTACACTTGAACGTATTGAGAAGAACTTTGTCATTACTGACCCAAGATTGCCAGATAATCCCATTATATTCGCGTCCGATAGTTTCTTGCAGTTGACAGAATATAGCCGTGAAGAAATTTTGGGAAGAAACTGCAGGTTTCTACAAGGTCCTGAAACTGATCGCGCGACAGTGAGAAAAATTAGAGATGCCATAGATAACCAAACAGAGGTCACTGTTCAGCTGATTAATTATACAAAGAGTGGTAAAAAGTTCTGGAACCTCTTTCACTTGCAGCCTATGCGAGATCAGAAGGGAGATGTCCAGTACTTTATTGGGGTTCAGTTGGATGGAACTGAGCATGTCCGAGATGCTGCCGAGAGAGAGGGAGTCATGCTGATTAAGAAAACTGCAGAAAATATTGATGAGGCGGCAAAAGAACTTGGCGGTGGCGGATCCGGAGGCGGCGGTTCCGGAGGCGGCGGAATGAGGGGGATGCTGCCCCTCTTTGAGCCCAAGGGCCGGGTCCTCCTGGTGGACGGCCACCACCTGGCCTACCGCACCTTCCACGCCCTGAAGGGCCTCACCACCAGCCGGGGGGAGCCGGTGCAGGCGGTCTACGGCTTCGCCAAGAGCCTCCTCAAGGCCCTCAAGGAGGACGGGGACGCGGTGATCGTGGTCTTTGACGCCAAGGCCCCCTCCTTCCGCCACGAGGCCTACGGGGGGTACAAGGCGGGCCGGGCCCCCACGCCGGAGGACTTTCCCCGGCAACTCGCCCTCATCAAGGAGCTGGTGGACCTCCTGGGGCTGGCGCGCCTCGAGGTCCCGGGCTACGAGGCGGACGACGTCCTGGCCAGCCTGGCCAAGAAGGCGGAAAAGGAGGGCTACGAGGTCCGCATCCTCACCGCCGACAAAGACCTTTACCAGCTCCTTTCCGACCGCATCCACGTCCTCCACCCCGAGGGGTATCTCATCACCCCGGCCTGGCTTTGGGAAAAGTACGGCCTGAGGCCCGACCAGTGGGCCGACTACCGGGCCCTGACCGGGGACGAGTCCGACAACCTTCCCGGGGTCAAGGGCATCGGGGAGAAGACGGCGAGGAAACTTCTGGAGGAGTGGGGGAGCCTGGAAGCCCTCCTCAAGAACCTGGACCGGCTGAAGCCCGCCATCCGGGAGAAGATCCTGGCCCACATGGACGATCTGAAGCTCTCCTGGGACCTGGCCAAGGTGCGCACCGACCTGCCCCTGGAGGTGGACTTCGCCAAAAGGCGGGAGCCCGACCGGGAGAGGCTTAGGGCCTTTCTGGAGAGGCTTGAGTTTGGCAGCCTCCTCCACGAGTTCGGCCTTCTGGAAAGCCCCAAGGCCCTGGAGGAGGCCCCCTGGCCCCCGCCGGAAGGGGCCTTCGTGGGCTTTGTGCTTTCCCGCAAGGAGCCCATGTGGGCCGATCTTCTGGCCCTGGCCGCCGCCAGGGGGGGCCGGGTCCACCGGGCCCCCGAGCCTTATAAAGCCCTCAGGGACCTGAAGGAGGCGCGGGGGCTTCTCGCCAAAGACCTGAGCGTTCTGGCCCTGAGGGAAGGCCTTGGCCTCCCGCCCGGCGACGACCCCATGCTCCTCGCCTACCTCCTGGACCCTTCCAACACCACCCCCGAGGGGGTGGCCCGGCGCTACGGCGGGGAGTGGACGGAGGAGGCGGGGGAGCGGGCCGCCCTTTCCGAGAGGCTCTTCGCCAACCTGTGGGGGAGGCTTGAGGGGGAGGAGAGGCTCCTTTGGCTTTACCGGGAGGTGGAGAGGCCCCTTTCCGCTGTCCTGGCCCACATGGAGGCCACGGGGGTGCGCCTGGACGTGGCCTATCTCAGGGCCTTGTCCCTGGAGGTGGCCGAGGAGATCGCCCGCCTCGAGGCCGAGGTCTTCCGCCTGGCCGGCCACCCCTTCAACCTCAACTCCCGGGACCAGCTGGAAAGGGTCCTCTTTGACGAGCTAGGGCTTCCCGCCATCGGCAAGACGGAGAAGACCGGCAAGCGCTCCACCAGCGCCGCCGTCCTGGAGGCCCTCCGCGAGGCCCACCCCATCGTGGAGAAGATCCTGCAGTACCGGGAGCTCACCAAGCTGAAGAGCACCTACATTGACCCCTTGCCGGACCTCATCCACCCCAGGACGGGCCGCCTCCACACCCGCTTCAACCAGACGGCCACGGCCACGGGCAGGCTAAGTAGCTCCGATCCCAACCTCCAGAACATCCCCGTCCGCACCCCGCTTGGGCAGAGGATCCGCCGGGCCTTCATCGCCGAGGAGGGGTGGCTATTGGTGGCCCTGGACTATAGCCAGATAGAGCTCAGGGTGCTGGCCCACCTCTCCGGCGACGAGAACCTGATCCGGGTCTTCCAGGAGGGGCGGGACATCCACACGGAGACCGCCAGCTGGATGTTCGGCGTCCCCCGGGAGGCCGTGGACCCCCTGATGCGCCGGGCGGCCAAGACCATCAACTTCGGGGTCCTCTACGGCATGTCGGCCCACCGCCTCTCCCAGGAGCTAGCCATCCCTTACGAGGAGGCCCAGGCCTTCATTGAGCGCTACTTTCAGAGCTTCCCCAAGGTGCGGGCCTGGATTGAGAAGACCCTGGAGGAGGGCAGGAGGCGGGGGTACGTGGAGACCCTCTTCGGCCGCCGCCGCTACGTGCCAGACCTAGAGGCCCGGGTGAAGAGCGTGCGGGAGGCGGCCGAGCGCATGGCCTTCAACATGCCCGTCCAGGGCACCGCCGCCGACCTCATGAAGCTGGCTATGGTGAAGCTCTTCCCCAGGCTGGAGGAAATGGGGGCCAGGATGCTCCTTCAGGTCCACGACGAGCTGGTCCTCGAGGCCCCAAAAGAGAGGGCGGAGGCCGTGGCCCGGCTGGCCAAGGAGGTCATGGAGGGGGTGTATCCCCTGGCCGTGCCCCTGGAGGTGGAGGTGGGGATAGGGGAGGACTGGCTCTCCGCCAAGGAG

LOV2-Adenylate kinase

GAAATGAGAAAGGGTATTGATCTAGCTACTACACTCGAACGTATCGAGAAGAATTTCGTCATCACTGATCCTAGGCTTCCCGATAATCCCATTATTTTTGCATCTGATAGTTTCTTGGAGCTCACGGAATATAGCCGTGAAGAAATTCTTGGAAGAAATTGCAGGTTTCTACAAGGTCCAGAGACTGATCTAACCACAGTGAAGAAGATTCGAAATGCTATTGATAACCAAACCGAAGTGACAGTTCAGCTCATCAACTACACCAAGAGCGGAAAGAAGTTCTGGAACATTTTCCACTTGCAACCTATGCGTGATCAGAAGGGAGAAGTACAATACTTTATTGGAGTTCAACTAGACGGGAGCAAGCACGTAGAACCAGTTCGCGGGAGCATGCGTATCATTCTGCTTGGCGCTCCGGGCGCGGGGAAAGGGACTCAGGCTCAGTTCATCATGGAGAAATATGGTATTCCGCAAATCTCCACTGGCGATATGCTGCGTGCTGCGGTCAAATCTGGCTCCGAGCTGGGTAAACAAGCAAAAGACATTATGGATGCTGGCAAACTGGTCACCGACGAACTGGTGATCGCGCTGGTTAAAGAGCGCATTGCTCAGGAAGACTGCCGTAATGGTTTCCTGTTGGACGGCTTCCCGCGTACCATTCCGCAGGCAGACGCGATGAAAGAAGCGGGCATCAATGTTGATTACGTTCTGGAATTCGACGTACCGGACGAACTGATCGTTGACCGTATCGTCGGTCGCCGCGTTCATGCGCCGTCTGGTCGTGTTTATCACGTTAAATTCAATCCGCCGAAAGTAGAAGGCAAAGACGACGTTACCGGTGAAGAACTGACTACCCGTAAAGATGATCAGGAAGAGACCGTACGTAAACGTCTGGTTGAATACCATCAGATGACAGCACCGCTGATCGGCTACTACTCCAAAGAAGCAGAAGCGGGTAATACCAAATACGCGAAAGTTGACGGCACCAAGCCGGTTGCTGAAGTTCGCGCTGATCTGGAAAAAATCCTCGGC

Oligonucleotide sequences:

University of Wuerzburg Light gated polymerase

GATCTCCTGGTACTACACTGACTGTAGGACTGTGCTATGGATCTACACGCACATGATCACGTCATCCTGGTCCGTTAGGTCACTTAGCTATACGAGTGTATCGACACACTACACACTTGACTA

Wuerzburg

ATGGATCTACACGCACATGATCACGT

# Supplementary Figures and Tables

## Supplementary Figures


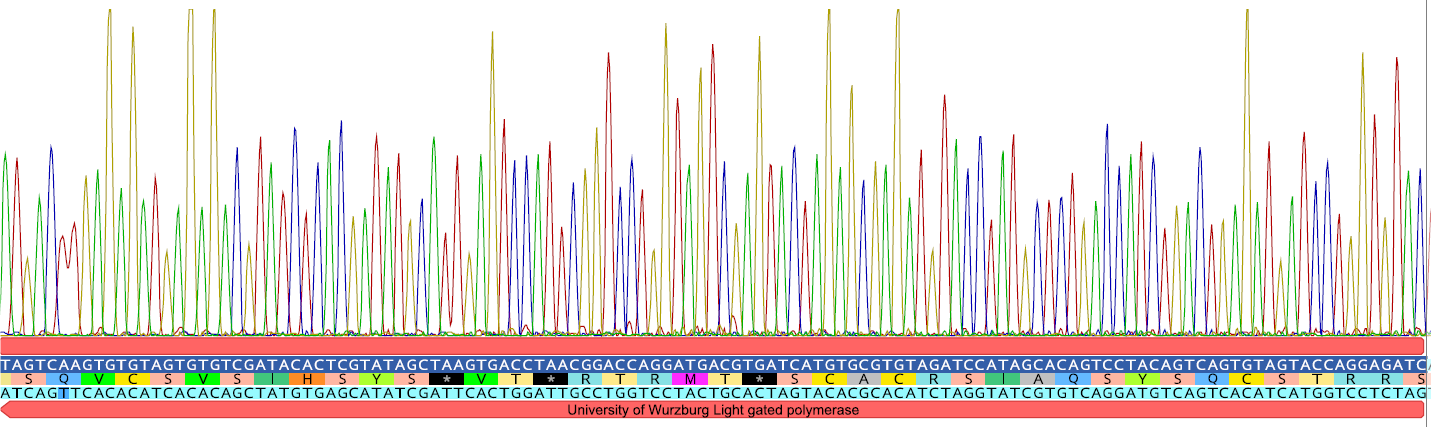


**Supplementary Figure 1.** Sequenced DNA after 2 years on the dried nanocellulose. Nanocellulose with DNA was hydrated and sequenced by Sanger sequencing.


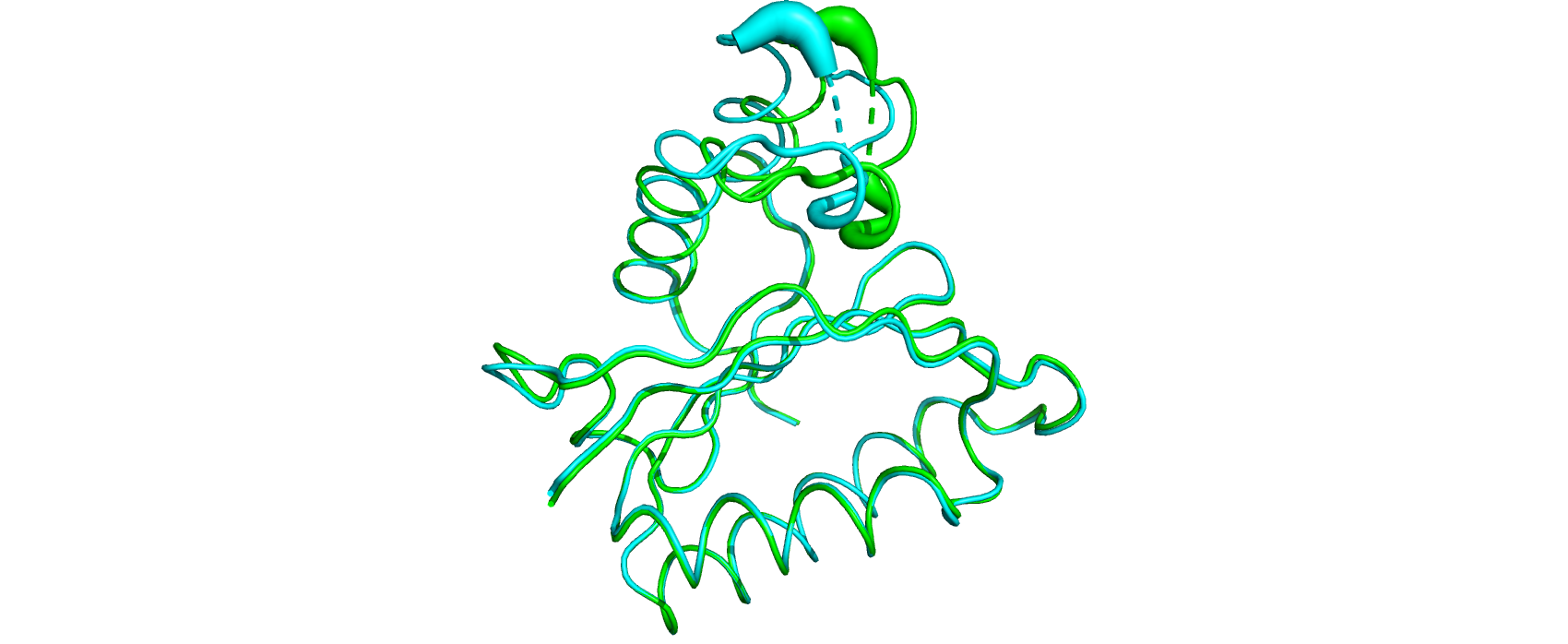


**Supplementary Figure 2:** Comparing the BLUF domain of BlsA at the ground (green) and photo-activated states (cyan). The proteins and their PPI regions are visualized as cartoon loop and putty models, respectively.  We show now: its conformational energy should be high enough due to its photo activation. The BLUF-POL interaction hypothesis is photo-activated BLUF domain  (its activated conformation) has a higher affinity to the DNA-POL, when it is inactive the affinity decreases, etc. The conformational shift was evaluated *in silico*. We have used the BLUF domain of BlsA at the ground (green) and photo-activated states (cyan). As the reader can see, there is a conformational shift of the flexible loop structure upon the BLUF activation (RMSD = 1.76 A) in the residues 110-122 (with a gap: 113 and 114 aa). These residues were predicted as a protein-protein interface to be most likely involved in the interaction with polymerase or T4 polynucleotide kinase. An elevated energy level was also detected starting from -2185.69 kcal/mol for the ground state to -2051.68 kcal/mol for the activated state. This mechanism might explain how a BLUF domain interacts with the polymerase or activate it. Similarly, this helps to clarify how a BLUF domain stops or activates the T4 polynucleotide kinase.

## Supplementary Tables

| Type of construct/ oligonucleotide | Name/Sequence | Reference |
| --- | --- | --- |
| Synthetic construct for read-out | BLUF-Exonuclease (sequence in the supplementary material) | This paper |
| Synthetic construct for phosphorylation of individual letters | LOV2-Adenylate kinase (sequence in the supplementary material) | This paper |
| Synthetic construct for read-in | LOV-Taq polymerase (sequence in the supplementary material) | This paper |
| Synthetic construct for digital writing | BLUF-Adenyltransferase (sequence in the supplementary material) | This paper |
| Synthetic construct for phosphorylation of a nucleotide sequence | BLUF-T4 kinase (sequence in the supplementary material) | This paper |
| Synthetic construct for RNA read-in | BLUF-Cid1 polymerase (including the mutated H336D and H336A version, sequences in the supplementary material) | This paper |

**Table S1**: List of available light-gated protein constructs used in our investigation

| DNA storage:  Longer Oligo | University of Wuerzburg Light gated polymerase (sequence in the supplementary material) | This paper |
| --- | --- | --- |
| DNA storage:  Shorter Oligo | Wuerzburg (sequence in the supplementary material) | This paper |
| Self-assembly oligonucleotides:  These assemble by themselves to DNA Macramée; | 1. GGAAAATTTGGAGA 2. GGAAACGTTGGAGA 3. GGAAAGCTTGGAGA 4. GGAAATATTGGAGA | Saoji and Paukstelis, 2015 |
| DNA quadruplex | 1. TGTGTGTGTGTGTGTGTGTG 2. TGTGTGTGTGTGTGTGTGTGTGTGTGTGTGTGTGTGTGTGTGTGTGTGTG | Dvorkin et al., 2018 |
| DNA quadruplex | 1. TTTTGGGGTTTT 2. TTTTGGGGGGGGGGGGTTTT | Livshits et al., 2014 |

**Table S2:** List of the oligonucleotides used for DNA storage and other applications described in the paper

**References:**

Saoji, M., & Paukstelis, P. J. (2015). Sequence-dependent structural changes in a self-assembling DNA oligonucleotide. *Acta Crystallographica Section D: Biological Crystallography*, *71*(12), 2471-2478.

Dvorkin, S. A., Karsisiotis, A. I., & Webba da Silva, M. (2018). Encoding canonical DNA quadruplex structure. *Science advances*, *4*(8), eaat3007.

Livshits, G. I., Stern, A., Rotem, D., Borovok, N., Eidelshtein, G., Migliore, A., ... & Porath, D. (2014). Long-range charge transport in single G-quadruplex DNA molecules. *Nature nanotechnology*, *9*(12), 1040-1046.
